# Evidence-based brief cessation advice plus active referral for emergency department patients who smoke: a single-arm, real-world clinical trial

**DOI:** 10.1186/s12916-025-04534-9

**Published:** 2025-11-27

**Authors:** Ho Cheung William Li, Wei Xia, Lishan Li, Hong Chen, Laurie Long Kwan Ho, Kai Yeung Cheung, Sarah Xiao, Yiu Cheung Chan, Oi Kwan Joyce Chung

**Affiliations:** 1 The Nethersole School of Nursing, The Chinese University of Hong Kong, Hong Kong SAR, China; 2 School of Nursing, Sun Yat-Sen University, Guangzhou, China; 3 School of Nursing, The Hong Kong Polytechnic University, Hong Kong SAR, China; 4 Accident and Emergency Department, United Christian Hospital, Hong Kong SAR, China

**Keywords:** Smoking cessation, Emergency department, Brief advice, Active referral, Health care professionals, Clinical trial, Real-world settings

## Abstract

**Background:**

Emergency department (ED) visits present opportunities to promote smoking cessation, as patients seeking urgent care may be more receptive to adopting healthier behaviors. This study evaluated the efficacy of brief cessation advice plus active referral to smoking cessation services among smokers attending EDs.

**Methods:**

A single-arm, real-world, multicenter clinical trial was conducted in the EDs of four public hospitals in Hong Kong from August 2019 to November 2021. Current smokers aged ≥ 18 years, triaged as semi- or non-urgent, were enrolled. Participants received the AWARD (Ask, Warn, Advise, Refer, and Do-it-again) model-based brief counseling and were offered active referral by trained healthcare professionals (HCPs) to local cessation services. Booster calls at 1 week and 1 month reinforced referrals for those who accepted. All participants were followed up at 6 and 12 months. The primary outcome was biochemically validated 7-day point prevalence abstinence (PPA) at 6 months; secondary outcomes included validated PPA at 12 months, self-reported PPA, and cigarette reduction rates. Implementation outcomes were evaluated using the RE-AIM framework. Propensity score matching (PSM) and multivariable logistic regression were applied to compare referred and unreferred groups.

**Results:**

Among the 1601 enrolled smokers, most were male (1443, 90%), with a mean age of 48 years; 77.6% and 72.1% completed 6-month and 12-month follow-ups, respectively. Following the brief advice intervention at baseline, 455 (28.4%) participants opted to receive a referral to smoking cessation services. Their overall biochemically validated 7-day PPA was 4.4% and 5.1% at 6 and 12 months, respectively. After PSM, the active referral group had significantly higher biochemically validated 7-day PPA at 6 months (6.1% vs. 3.2%; adjusted OR 2.35, *p* = .009) and 12-month follow-up (9.3% vs. 3.0%; adjusted OR 3.54, *p* < .001), higher self-reported 7-day PPA, and significant reduction rates at 6 and 12 months compared with the unreferred group. Implementation outcomes revealed strong institutional adoption, with 93 trained HCPs and a high reach among eligible smokers. This trial was limited by a non-randomized design.

**Conclusions:**

Brief cessation advice combined with active referral is feasible in ED settings and may yield clinically meaningful improvements in smoking abstinence.

**Trial registration:**

ClinicalTrials.gov: NCT03818360, 25 January 2019.

**Supplementary Information:**

The online version contains supplementary material available at 10.1186/s12916-025-04534-9.

## Contributions to the literature

- Emergency departments (EDs) visits offer a unique opportunity for smoking cessation efforts; however, comprehensive interventions are often impractical in busy EDs, and effective, practical interventions are lacking.
- Brief advice plus active referral to stop smoking services was cost-effective and feasible to be implemented in ED settings and may support clinically meaningful quit rates among patients who smoke.
- The findings contribute to the literature on effective smoking cessation interventions that can be integrated into routine clinical practice with minimal disruption and the potential to be scaled up in clinical settings.

## Background

Smoking exerts harmful effects on nearly every organ of the body and is responsible for eight million deaths annually worldwide [1]. Emergency departments (EDs) function as locations for acute medical emergencies and provide health-care access to patients lacking a primary care provider [2–4]. In addition, people who smoke tend to visit EDs at a disproportionately higher rate than the general population [5, 6]. Seeking emergency help may heighten smokers’ motivation to adopt healthier behaviors, including quitting smoking [7, 8]. Therefore, ED visits represent an excellent “teachable moment” to address these patients’ tobacco use and encourage smoking cessation, particularly for people who may experience barriers to accessing routine health care due to the social determinants of health disparities, making this one of the few moments they come into contact with health-care professionals (HCPs).

The results of three systematic reviews and meta-analyses demonstrated the effectiveness of comprehensive smoking cessation interventions initiated in EDs, such as motivational interviewing counseling, pharmacotherapy, and ongoing support [9–11]. Nicotine replacement therapy (NRT) and electronic cigarettes (EC) have also been frequently used in smoking cessation interventions. While studies have proven that NRT could help improve smoking cessation rates [12], cost and lack of insurance coverage can limit access to evidence-based cessation treatments, including NRT [13]. Regarding ECs, a recent network meta-analysis of 319 randomized controlled trials provided high-certainty evidence that nicotine ECs are more effective in promoting smoking cessation than non-pharmacological interventions or placebo [14]. Another Cochrane systematic review found high-certainty and moderate-certainty evidence that nicotine ECs increase quit rates compared to NRT and non-nicotine ECs, respectively. However, the evidence was of low certainty when compared with usual care or no treatment, due to the risk of bias and the limited number of trials [15]. In terms of safety, current evidence does not suggest serious short-term harm when ECs are used for smoking cessation, although the certainty of safety outcomes remains low and longer-term studies are needed [14, 15]. Specifically, one and two trials reported significant intervention benefits at 1 and 3 months, respectively [9–11]; however, few trials have evaluated biochemically validated abstinence in long-term follow-up of at least 6 months. Additionally, delivering intensive behavioral interventions is often not feasible in busy ED settings [16, 17]. Lack of time, training, confidence in the intervention, and support from hospital management are the main barriers reported by HCPs in their efforts to help individuals quit smoking [18, 19]. Our previous smoking cessation trials revealed that many patients were reluctant to participate in more intensive interventions lasting 30–45 min during their ED visit, due to impatience or concerns about missed or delayed medical procedures [20, 21].

A recent trial conducted among daily smokers visiting an ED in the United Kingdom showed that interventions comprising brief advice, an EC starter kit, and referrals to smoking cessation services were effective for increasing sustained biochemically validated abstinence at the 6-month follow-up, compared with signposting smoking cessation services [22, 23]. Similarly, we previously conducted an RCT in multiple EDs using the ask, warn, advise, refer, and do-it-again (AWARD) model, in addition to a brief smoking cessation intervention based on self-determination theory, which demonstrated increased quit rates at 6 and 12 months [24]. These results indicate that after minimal training, a brief intervention would be cost-effective and feasible for routine use by HCPs in clinical practice. However, the effectiveness of these brief interventions in real-world settings is still unknown, as patients are managed in these settings with minimal study-related restrictions, which allow for the flexible selection of specific patient subgroups and diverse care locations, as opposed to the strictly controlled contexts and random assignments used in RCTs [25]. Moreover, brief interventions in clinical settings may fail to ensure long-term abstinence without follow-up, especially among heavy smokers [26]. However, smoking cessation services have been shown to effectively support individuals in their efforts to quit smoking [27, 28]. Therefore, providing advice and referrals to smoking cessation services could improve intervention outcomes for patients who need extra support [29, 30]. Notably, a recent interview exploring barriers to engagement with smoking cessation interventions in EDs, such as lack of contact or misalignment of services with participants’ needs, highlighted the need for innovative approaches, such as active referral, to enhance service utilization.

Although the prevalence of daily cigarette smoking in Hong Kong decreased from 23.3% in 1982 to 9.1% in 2024, there are still 581,500 daily smokers in Hong Kong, which is associated with 400,000 annual hospitalizations [31]. Moreover, 31.2% of cigarette smokers in Hong Kong have tried to quit smoking but failed. In addition, smoking cessation services are generally poorly used, with a utilization rate of 23.2% [31]. Considering the health needs of the smoking community, we proposed an intervention combining brief advice offered by HCPs with minimal training and the provision of referrals to smoking cessation services with follow-up to achieve a greater intervention effect.

In the current study, we aimed to evaluate the efficacy of the proposed brief cessation advice plus active referral intervention among smokers visiting EDs and its feasibility for implementation in real-world clinical settings. We hypothesized that brief cessation advice plus active referral would result in higher abstinence rates than brief advice alone.

## Methods

### Study design and setting

We conducted a single-arm multicenter clinical trial in the EDs of four hospitals in Hong Kong from 30 August 2019 to 26 November 2021, which coincided with the outbreak of the COVID-19 pandemic. This pragmatic research was conducted in real-world settings in which patients were waiting to receive treatment from ED doctors whose evaluations were guided by the reach, effectiveness, adoption, implementation, and maintenance (RE-AIM) framework [32]. The trial was prospectively registered in ClinicalTrials.gov (ID: NCT03818360), and its protocol is presented in Additional file 1: Protocol. The Transparent Reporting of Evaluations with Non-randomized Designs (TREND) reporting guideline was followed (see Additional file 2: TREND Statement Checklist) [33]. Ethical approval was obtained from the Institutional Review Board of the University of Hong Kong/Hospital Authority Hong Kong West Cluster (UW19-032) and the enrolled hospitals.

### Participants

#### Health care professionals (HCPs)

We sent invitation letters to five of the 16 EDs in districts managed by the Hong Kong Hospital Authority. Four EDs agreed to participate and were asked to select approximately 10 HCPs each to attend a half-day training session organized by the research team to ensure the continued implementation of the intervention.

From 13 August 2019 to 17 December 2019, 93 HCPs from the four participating EDs completed the training workshop. The HCPs’ training content focused on (1) smoking and health, smoking cessation services, and tobacco control policies in Hong Kong, (2) a brief smoking cessation intervention based on the AWARD model to advise smokers to quit immediately or quit progressively over an acceptable period, and (3) procedures for referring smokers to smoking cessation services. Follow-up interviews were conducted immediately after the training and at 3 and 6 months using structured questionnaires (see Additional file 3: Questionnaires-Healthcare Professionals).

#### Participants who smoke

Inclusion criteria for participants presenting in the selected EDs were as follows: (1) aged 18 years or older, (2) triaged as semi- or non-urgent, (3) current smokers averaging at least one cigarette per day or equivalent. Participants were excluded if they had (1) impaired mental status or cognitive impairment, (2) communication barriers; or (3) participated in other smoking cessation programs or services.

The sample size was estimated based on our previous RCT of a brief advice intervention in EDs, which indicated 6-month biochemically validated quit rates of 6.7% and 2.8% in the intervention and control groups, respectively [24]. In the pilot study, the ratio was approximately 1:2 for accepting and refusing referrals, respectively. To achieve a power of 80% and a two-sided significance level of 5%, this study required a sample size of 1098. Following our previous experience, the retention rate at the 12-month follow-up was approximately 70%; thus, at least 1569 participants were needed in this study.

### Intervention

The eligible participants received either brief cessation advice plus active referral (i.e., referred group) or brief advice only (i.e., unreferred group) based on their agreement to be referred to smoking cessation services in Hong Kong.

#### Referred group

The participants who agreed to be included and were referred to smoking cessation services received a leaflet about the hazards of smoking, directions to stop-smoking services, brief counseling using the AWARD model delivered by trained HCPs, an offer of active referral to smoking cessation services, and booster follow-up phone calls at 1 week and 1 month to enhance and confirm the referral.

#### Unreferred group

The participants who were not referred to smoking cessation services received the same protocol as those in the referred group, except they did not receive a referral to smoking cessation services or booster follow-up phone calls.

### Procedures

A clinical nurse triaged individuals visiting the ED as critical, emergency, urgent, semi-urgent, or non-urgent [34]. A research nurse then proactively approached and screened these individuals during their wait for a doctor to identify eligible smokers [35]. All smokers visiting the EDs promptly received a leaflet about the health-related hazards of smoking and benefits of quitting as well as an information card containing brief information about the existing smoking cessation services in Hong Kong (e.g., smoking cessation telephone hotlines, addresses, and operational hours) from the triage nurses, who then referred the participants to trained HCPs. These HCPs engaged in brief counseling using the five components of the AWARD model, which can be effectively administered in a concise timeframe: (1) ask about their smoking history, (2) warn about the high risk of smoking, (3) advise smokers to quit as soon as possible and comply with their decided quit date, (4) refer smokers to smoking cessation services, and (5) do it again [24]. In the fourth step “Refer”, the HCPs described existing smoking cessation services to the study participants and asked them to indicate their preferred smoking cessation service and provide their informed consent and contact information to the project team. Participants who agreed to be referred and provided contact information were actively referred, and their contact details were emailed to their chosen service provider within 1 week of recruitment. Within the week, smokers received proactive telephone calls from their chosen smoking cessation service provider and were offered cessation counseling or to book an appointment at the smoking cessation clinic.

Booster follow-up calls by the research team at 1 week and 1 month after baseline were conducted among the participants who agreed to be referred but could not be reached by smoking cessation services. Multiple attempts were made to contact these participants to ensure their contact information was accurate and to prevent errors in communication during active referral, without the evaluation of outcomes.

Regardless of referral status, all participants were followed up again at 6 and 12 months using telephone interviews for data collection.

#### Measures and outcomes

Following the RE-AIM evaluation framework, the efficacy assessment included an evaluation of primary effectiveness (i.e., patient-reported outcomes) and implementation outcomes [32]. The participants’ baseline data, including their demographic characteristics and smoking profiles, were obtained in person using a structured questionnaire (see Additional file 4: Questionnaires-Patients) based on previous trials [20, 21, 24]. Follow-up telephone evaluations were conducted at 6 and 12 months.

The primary outcome of effectiveness was biochemically validated 7-day point prevalence abstinence (PPA) at 6 months, as this method is objective, sensitive to the intervention, and widely used in smoking cessation trials to detect early effectiveness. Biochemically validated PPA was defined as exhaled carbon monoxide levels < 4 parts per million (ppm) and salivary cotinine levels < 10 ng/mL, following the recommendations of the Society of Research on Nicotine and Tobacco [36]. Participants who self-reported quitting in the past 7 days at the 6-month follow-up were invited to participate in a biochemical validation test and then were informed that a 38 USD cash payment covering their time and traveling expenses would be offered, which has been shown to have no impact on smoking cessation rates [37, 38].

The secondary outcomes included (1) biochemically validated 7-day PPA at 12 months for assessing long-term effectiveness; (2) self-reported 7-day PPA from all tobacco products, including traditional cigarettes and other tobacco products containing nicotine (e.g., ECs, shisha, cigars, rolled cigarettes, tobacco pipes, heat-not-burn tobacco products) at 6 and 12 months; (3) self-reported smoking reduction at 6 and 12 months, defined as a reduction of daily cigarette consumption from baseline by at least 50%. We also assessed self-reported 7-day PPA from traditional cigarettes, the use of other tobacco products, nicotine dependency level, and the stage of readiness to quit as secondary outcomes.

We used the RE-AIM indexes (See Additional files 5: Table S1) to assess the implementation outcomes, including reach, efficacy, adoption, implementation, and maintenance. The schedule of assessments was presented in Additional file 6: Table S2.

#### Statistical analysis

IBM SPSS software (v. 26; IBM SPSS, Armonk, NY, USA) was used for the data analysis. Following the Russell standard, the smoking outcomes in which participants were lost to follow-up or refused to participate in the validation tests were conservatively considered smokers, with no reduction in cigarette consumption compared with baseline based on the intention-to-treat (ITT) method [39, 40].

We conducted χ^2^ tests for categorical variables and *t*-tests for continuous variables using both matched datasets to compare the baseline information and outcomes between the two participant groups. Odds ratios (ORs) and 95% confidence intervals (CIs) for primary and secondary outcomes were calculated using multivariable logistic regressions, adjusting for potential imbalances in baseline characteristics. Sensitivity analyses for abstinence outcomes were conducted using generalized estimating equation (GEE) models, complete case analysis, and multiple imputed outcomes for the participants who were lost to follow-up or who refused to participate in the validation tests. Missing values were handled by multiple imputation using the Markov chain Monte Carlo method to generate 10 datasets with imputed missing data [41–43]. The imputation model included socio-demographic characteristics and smoking-related variables at baseline and outcomes at all follow-ups, including age, hospital, employment status, annual income level, level of nicotine dependence, readiness to quit, smoking self-efficacy, validated 7-day PPA, and self-reported 7-day PPA, with two-way interactions among categorical predictors. These variables were also included in the model to reduce bias and incompatibility. Regression parameters and the corresponding standard errors of all imputed datasets were pooled using Rubin’s rules [44].

To minimize the effects of potential confounding factors on the primary and secondary outcome measures, we conducted a two-way propensity score matched (PSM) analysis between the referred and unreferred groups, including all demographic and smoking variables in the multinomial regression, to maximally inform the propensity of the dependent variables [45]. The referred and unreferred groups were matched 1:2 using a nearest-neighbor approach with caliper restrictions (see Additional file 7: Table S3) [46, 47].

To confirm the overall efficacy of this real-world intervention, a three-way PSM analysis including participants in the current study (i.e., brief advice and active referral) as well as the intervention group (i.e., brief advice and self-determination to quit immediately or progressively) and control group (i.e., smoking cessation leaflet and placebo treatment) in our previous RCT was performed with a matching ratio of 1:1:1 [24]. Standardized differences in the demographic and smoking variables were compared with diagnoses to balance the matched groups (see Additional file 8: Table S4 and Additional file 9: Table S5) [48]. A similar analysis approach based on ITT was conducted to explore the differences in primary and secondary outcomes using the three-way PSM dataset. A brief cost-effectiveness analysis was performed to estimate the incremental cost-effectiveness ratio (ICER) of the active referral provided by HCPs.

## Results

**Fig. 1:**
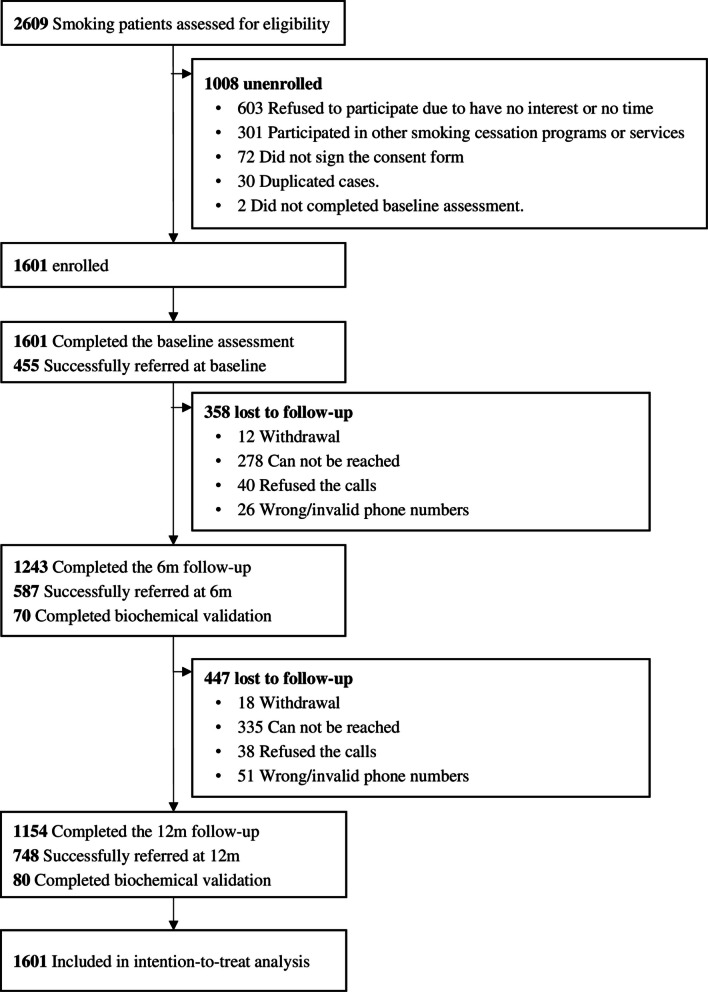
Trial flow diagram

From 30 August 2019 to 26 November 2021, 2609 of the 30,356 (8.6%, eligibility rate) screened patients visiting EDs were identified as eligible. A total of 1601 participants (61.4%) completed the baseline assessment after providing their informed consent and were included in the analysis. The study process is presented in Fig. 1. The retention rates were 77.6% and 72.1% at 6 and 12 months, respectively. Table 1 shows that 90.1% of the participants were men, with a mean (standard deviation) age of 48.3 (15.0) years. Most had no intention to quit smoking (88.9%), and nearly half had mild nicotine dependence (48.3%) at baseline. After receiving the brief advice intervention at baseline, 455 (28.4%) participants were referred successfully to a smoking cessation service. Some participants who agreed to be referred but were not successfully referred at baseline due to hospitalization or other personal issues were found to be successfully referred at the 6- (*n* = 132) and 12-month (*n* = 161) follow-ups, with cumulative referral rates of 36.1% and 46.7%, respectively. No significant differences in baseline characteristics were observed between the referred and unreferred groups after PSM (Table 1).

**Table 1:** Participants’ demographic and smoking profile at baseline and difference between those who were referred and those not referred to smoking cessation services

Variable		Unmatched cases			Matched cases		
	All (*N* = 1601)	Referred (*n* = 455)	Unreferred (*n* = 1146)	*P* value	Referred (*n* = 396)	Unreferred (*n* = 792)	*P* value
Age, years, range:18–92^a^	48.3 (15.0)	47.1 (14.0)	48.8 (15.4)	0.03	46.4 (13.6)	47.4 (14.9)	0.28
Sex				0.06			0.81
Male	1443 (90.1)	400 (87.9)	1043 (91.0)		347 (87.6)	690 (87.1)	
Female	158 (9.9)	55 (12.1)	103 (9.0)		49 (12.4)	102 (12.9)	
Educational attainment ^a^				0.003			0.27
Primary or below	270 (17.2)	61 (14.1)	209 (18.4)		66 (16.7)	125 (15.8)	
Secondary	1087 (69.3)	327 (75.7)	760 (66.9)		286 (72.2)	552 (69.7)	
Tertiary or above	211 (13.5)	44 (10.2)	167 (14.7)		44 (11.1)	115 (14.5)	
Marital status^a^				0.91			0.90
Single	453 (28.6)	125 (27.8)	328 (28.8)		116 (29.3)	229 (28.9)	
Married/cohabited	1031 (65.0)	294 (65.5)	737 (64.8)		253 (63.9)	514 (64.9)	
Separated/divorced/widowed	102 (6.4)	30 (6.7)	72 (6.3)		27 (6.8)	49 (6.2)	
Employment status ^a^				0.93			0.95
Student	23 (1.4)	7 (1.6)	16 (1.4)		8 (2.0)	18 (2.3)	
Employed	1156 (72.7)	325 (72.1)	831 (72.9)		279 (70.5)	560 (70.7)	
Unemployed or retired	412 (25.9)	119 (26.4)	293 (25.7)		109 (27.5)	214 (27.0)	
Daily traditional cigarette consumption, range:0–120	13.8 (9.3)	14.8 (8.8)	13.4 (9.4)	0.007	14.7 (8.7)	13.9 (9.3)	0.16
Regular tobacco use time, years, range:0–74	30.5 (15.5)	29.5 (14.5)	30.9 (15.9)	0.097	28.9 (14.1)	29.6 (15.2)	0.42
History of using other tobacco products ^a^	594 (37.1)	162 (35.6)	432 (37.7)	0.44	149 (37.6)	308 (38.9)	0.67
Currently using other tobacco products	179 (11.2)	44 (9.7)	135 (11.8)	0.23	40 (10.1)	89 (11.2)	0.55
Previous ever quit attempts ^a^	1062 (66.3)	316 (69.5)	746 (65.1)	0.10	276 (69.7)	553 (69.8)	0.96
Previous ever quit attempts within 1 year ^a^	320 (20.0)	104 (22.9)	216 (18.8)	0.07	90 (22.7)	169 (21.3)	0.59
Ever services used	303 (18.9)	137 (30.1)	166 (14.5)	< 0.001	100 (25.3)	166 (21.0)	0.09
Nicotine dependency by the FTND^b^				< 0.001			0.69
Mild, 0–3	772 (48.3)	186 (40.9)	586 (51.2)		176 (44.4)	367 (46.3)	
Moderate, 4–5	441 (27.5)	152 (33.4)	289 (25.2)		127 (32.1)	235 (29.7)	
Severe, 6–10	387 (24.2)	117 (25.7)	270 (23.6)		93 (23.5)	190 (24.0)	
Intention to quit ^a^				< 0.001			0.09
Pre-contemplation	1422 (88.9)	360 (79.1)	1064 (92.8)		338 (85.4)	710 (89.6)	
Contemplation	105 (6.6)	63 (13.8)	42 (3.7)		36 (9.1)	42 (5.3)	
Preparation	45 (2.8)	21 (4.6)	24 (2.1)		13 (3.3)	24 (3.0)	
Action	17 (1.7)	11 (2.4)	16 (1.4)		9 (2.3)	16 (2.0)	

*FTND* Fagerström Test for Nicotine Dependence

^a^With missing value

### Primary and secondary outcomes

Among smokers who self-reported quitting for 7 days, 70 (41.2%) and 80 (43.8%) were biochemically validated, with a similar proportion and pass rate between the referred and unreferred groups at 6 (40.0% vs. 41.9%; *P* = 0.81) and 12 months (36.9% vs. 28.1%, *P* = 0.14), respectively. As shown in Table 2, the overall primary outcome of biochemically validated 7-day PPA at the 6-month showed the same proportion of 4.1% in the ITT analysis before (65of 1601) and after (49 of 1188) two-way PSM, with 6.1% (24 of 396) in the referred group versus 3.2% (25 of 792) in the unreferred group (adjusted OR (aOR), 2.35; 95% CI, 1.24–4.46; *P* = 0.009) after PSM.

**Table 2:** Outcomes before and after using two-way PSM between referred and unreferred groups

	Unmatched cases/*N* (%)					Matched cases/*N* (%)						
Variable	All (*n* = 1601)	Referred (*n* = 455)	Unreferred (*n* = 1146)	*P* value	Adjusted OR (95% CI)^d^	*P* value	All (*n* = 1188)	Referred (*n* = 396)	Unreferred (*n* = 792)	*P* value	Adjusted OR (95% CI)^d^	*P* value
Biochemically validated abstinence												
6 m	65 (4.1)	24 (5.3)	41 (3.6)	0.121	1.88 (1.04, 3.41)	0.04	49 (4.1)	24 (6.1)	25 (3.2)	0.018	2.35 (1.24, 4.46)	0.009
12 m	77 (4.8)	37 (8.1)	40 (3.5)	< 0.001	2.98 (1.70, 5.19)	< 0.001	61 (5.1)	37 (9.3)	24 (3.0)	< 0.001	3.54 (1.94, 6.45)	< 0.001
Self-reported 7-day PPA of all tobacco products												
6 m	170 (10.6)	65 (14.3)	105 (9.2)	0.003	1.72 (1.17, 2.55)	0.006	133 (11.2)	62 (15.7)	71 (9.0)	0.001	2.01 (1.34, 3.02)	0.001
12 m	244 (15.2)	83 (18.2)	161 (14.0)	0.035	1.37 (1.01, 1.86)	0.047	202 (17.0)	83 (21.0)	119 (15.0)	0.010	1.50 (1.08, 2.07)	0.02
Self-reported reduction of ≥ 50% in cigarette consumption ^a^												
6 m	204 (14.3)	79 (20.3)	125 (12.0)	< 0.001	1.93 (1.34, 2.79)	< 0.001	150 (12.6)	72 (25.1)	78 (10.8)	< 0.001	2.16 (1.47, 3.17)	< 0.001
12 m	264 (19.5)	91 (24.5)	173 (17.7)	0.005	1.47 (1.05, 2.06)	0.026	145 (14.7)	65 (27.8)	80 (18.3)	0.005	1.73 (1.21, 2.46)	0.002
Self-reported 7-day PPA of traditional cigarette												
6 m	252 (15.7)	77 (16.8)	175 (15.3)	0.413	1.28 (0.91, 1.82)	0.16	185 (15.6)	74 (18.7)	111 (14.0)	0.036	1.53 (1.07, 2.20)	0.02
12 m	280 (17.5)	88 (19.3)	192 (16.8)	0.219	1.21 (0.86, 1.69)	0.27	223 (18.8)	88 (22.2)	135 (17.0)	0.031	1.34 (0.95, 1.89)	0.100
Attempt to quit smoking ^a^												
6 m	418 (29.2)	151 (38.7)	267 (25.6)	< 0.001	1.76 (1.32, 2.34)	< 0.001	330 (31.3)	144 (50.2)	186 (35.6)	< 0.001	2.06 (1.53, 2.78)	< 0.001
12 m	546 (40.2)	185 (49.7)	361 (36.9)	< 0.001	1.70 (1.28, 2.26)	< 0.001	347 (35.2)	151 (64.5)	196 (45.0)	< 0.001	1.63 (1.25, 2.13)	< 0.001
Currently using other tobacco products												
6 m	99 (6.2)	23 (5.1)	76 (6.6)	0.237	0.74 (0.39, 1.41)	0.35	49 (4.1)	11 (3.8)	38 (8.7)	0.057	0.34 (0.14, 0.79)	0.01
12 m	108 (6.7)	25 (5.5)	83 (7.2)	0.208	0.82 (0.46, 1.49)	0.52	79 (6.6)	21 (5.3)	58 (7.3)	0.188	0.38 (0.18, 0.93)	0.01
FTND decreasing ^a,b^												
6 m	260 (18.2)	81 (20.8)	179 (17.2)	0.118	1.07 (0.81, 1.42)	0.65	201 (19.1)	70 (24.4)	131 (25.1)	0.824	1.19 (0.87, 1.61)	0.28
12 m	262 (19.3)	84 (22.6)	178 (18.2)	0.068	1.05 (0.80, 1.39)	0.72	196 (19.9)	70 (29.9)	126 (28.9)	0.783	1.04 (0.72, 1.52)	0.82
TTM stage promotion ^a,b,c^												
6 m	75 (5.2)	41 (10.5)	34 (3.3)	< 0.001	2.43 (1.74, 3.39)	< 0.001	65 (6.2)	38 (11.5)	27 (3.7)	< 0.001	1.74 (1.32, 2.31)	< 0.001
12 m	219 (16.1)	81 (21.8)	138 (14.1)	0.001	2.36 (1.66, 3.34)	< 0.001	147 (14.9)	65 (27.8)	82 (18.8)	0.007	1.57 (1.17, 2.10)	0.002

Participants lost to follow-up were assumed to be active smokers with no changes in their habits at baseline

^a^ Quitters were excluded

^b^ Compared to that at baseline

^c^ TTM stage promotion refers to participants who advanced to a higher level of readiness to quit smoking according to the transtheoretical model (e.g., transitioning from pre-contemplation to contemplation/preparation/action, contemplation to preparation/action, or preparation to action)

^d^ Estimates from the multiple logistic/linear regression adjusted for age, gender, education level, marital status, employment, cigarette consumption per day, regular tobacco use time, nicotine dependence level by FTND, stage of readiness to quit, quit attempt, referral history, other tobacco use, psychological perspectives on importance, confidence, and difficulty of quitting smoking at baseline

The participants reported overall biochemically validated 7-day PPA at 12 months (4.8%, 77 of 1601 and 5.1%, 61 of 1188), self-reported 7-day PPA at 6 and 12 months (10.6%, 170 of 1601 and 11.2%, 133 of 1188), and at 6 and 12 months (15.2%, 244 of 1601 and 17.0%, 202 of 1188) at 12 months, before and after PSM, respectively. The biochemically validated 7-day PPA at the 12-month (aOR, 3.54; 95% CI, 1.94–6.45; *P* < 0.001), and self-reported 7-day PPA from all tobacco products at 6 months (aOR, 2.01; 95% CI, 1.34–3.02; *P* = 0.001) and 12 months (aOR, 1.50; 95% CI, 1.08–2.07; *P* = 0.02), were also significantly higher in the referred group versus the unreferred group (Table 2).

After excluding those who quit smoking, participants who still smoked reported overall self-reported reduction rates of 14.3% (204 of 1431) and 14.2% (150 of 1055) at 6 months and of 19.5% (264 of 1357) and 14.7% (145 of 986) at 12 months, a 24-h quit attempt of 29.2% (418 of 1431) and 31.3% (330 of 1055) at 6 months and of 40.2% (546 of 1357) and 35.2% (347 of 986) at 12 months, and improvement in levels of readiness to quit of 5.2% (75 of 1431) and 6.2% (65 of 1055) at 6 months and of 16.1% (219 of 1357) and 14.9% (147 of 986) at 12 months, before and after PSM, respectively (Table 2). The rates of self-reported reduction at 6 months (aOR, 2.16; 95% CI, 1.47–3.17; *P* < 0.001) and 12 months (aOR, 1.73; 95% CI, 1.21–2.46; *P* = 0.002), including a 24-h quit attempt at 6 (aOR, 2.06; 95% CI, 1.53–2.78; *P* < 0.001) and 12 months (aOR, 1.63; 95% CI, 1.25–2.13; *P* < 0.001), as well as improvement in the levels of readiness to quit at 6 (aOR, 1.74; 95% CI, 1.32–2.31; *P* < 0.001) and 12 months (aOR, 1.57; 95% CI, 1.17–2.10; *P* = 0.002), were also significantly higher in the referred group compared with the unreferred group (Table 2). The unmatched analysis showed comparable results to those with matched cases, except for self-reported 7-day PPA from traditional cigarettes at 6 months, as well as currently using other tobacco products at 6 and 12 months, which showed insignificant differences between the referred and unreferred groups.

The differences in abstinence outcomes based on GEE models, multiply imputed outcomes, and complete case analyses showed comparable results to those of the primary analyses (see Additional file 10: Table S6).

It is important to note that the observed differences in smoking abstinence rates may be influenced by the booster follow-up calls, which were provided only to participants who accepted the referral. Those who declined the referral did not receive these follow-up calls.

### Exploratory analysis

The exploratory analysis using two-way PSM cases showed comparable results to those with unmatched cases, except for self-reported 7-day PPA from traditional cigarettes at 6 months (aOR, 1.53; 95% CI, 1.07–2.20; *P* = 0.02) and currently using other tobacco products at the 6- (aOR, 0.34; 95% CI, 0.14–0.79; *P* = 0.01) and 12-month follow-ups (aOR, 0.38; 95% CI, 0.18–0.93; *P* = 0.01), which showed significant differences between the referred and unreferred groups (Table 2).

As shown in Table 3, the analysis using three-way PSM cases showed that biochemically validated abstinence at 6 (aOR, 2.67; 95% CI, 1.17–6.09; *P* = 0.02) and 12 months (aOR, 2.51; 95% CI, 1.26–4.99; *P* = 0.009), with self-reported 7-day PPA from traditional cigarettes at 6 (aOR, 2.50; 95% CI, 1.58–3.97; *P* < 0.001) and 12 months (aOR, 4.70; 95% CI, 2.97–7.45; *P* < 0.001), self-reported reduction ≥ 50% in cigarette consumption at 12 months (aOR, 1.99; 95% CI, 1.33–2.98; *P* = 0.001), 24-h quit attempt at 6 (aOR, 3.01; 95% CI, 2.23–4.07; *P* < 0.001) and 12 months (aOR, 5.39; 95% CI, 3.94–7.37; *P* < 0.001), and decreased nicotine dependency levels at 6 (aOR, 8.77; 95% CI, 5.28–14.56; *P* < 0.001) and 12 months (aOR, 9.68; 95% CI, 6.15–15.24; *P* < 0.001) were significantly higher in the current study versus the control group in our previous RCT.

**Table 3:** Outcomes before and after using three-way PSM among Referral, Self-Quit, and Control groups

Unmatched cases	*N* (%)				Adjusted OR (95% CI)^a,b^	*P* value	Adjusted OR (95% CI)^a,c^	*P* value
	All (*n* = 3172)	Referral (*n* = 1601)	Self-Quit (*n* = 787)	Control (*n* = 784)				
Biochemically validated abstinence								
6 m	140 (4.4)	65 (4.1)	53 (6.7)	22 (2.8)	0.73 (0.44, 1.23)	0.24	1.85 (1.06, 3.23)	0.03
12 m	161 (5.1)	77 (4.8)	55 (7.0)	29 (3.7)	1.06 (0.65, 1.74)	0.81	1.90 (1.14, 3.16)	0.01
Self-reported 7-day PPA of traditional cigarette								
6 m	339 (10.7)	170 (10.6)	96 (12.0)	73 (9.3)	1.25 (0.87, 1.82)	0.23	1.64 (1.16, 2.30)	0.005
12 m	419 (13.2)	250 (15.6)	102 (13.0)	67 (8.5)	1.85 (1.31, 2.60)	< 0.001	2.83 (2.02, 3.95)	< 0.001
Self-reported reduction of ≥ 50% in cigarette consumption ^f^								
6 m	454 (16.0)	204 (14.3)	123 (17.8)	127 (17.9)	1.01 (0.72, 1.43)	0.94	0.81 (0.61,1.09)	0.16
12 m	499 (18.1)	264 (19.5)	130 (19.0)	105 (14.6)	1.18 (0.85, 1.64)	0.33	1.64 (1.22, 2.21)	0.001
Quit attempts ^d^								
6 m	1044 (36.9)	588 (36.7)	230 (29.2)	226 (28.8)	2.26 (1.74, 2.93)	< 0.001	2.20 (1.75, 2.77)	< 0.001
12 m	1221 (44.4)	796 (49.7)	203 (25.8)	222 (28.3)	5.32 (4.02, 7.04)	< 0.001	4.13 (3.27, 5.22)	< 0.001
Decreasing nicotine dependency by the FTND^d^								
6 m	500 (17.6)	385 (24.0)	61 (7.8)	54 (6.9)	5.27 (3.59, 7.71)	< 0.001	5.74 (4.00, 8.23)	< 0.001
12 m	672 (24.4)	551 (34.4)	58 (7.4)	63 (8.0)	8.45 (5.90, 12.32)	< 0.001	7.64 (5.45, 10.72)	< 0.001
Matched cases	All (*n* = 1284)	Referral (*n* = 428)	Self-Quit (*n* = 428)	Control (*n* = 428)	Adjusted OR (95% CI)^a,b^	*P* value	Adjusted OR (95% CI)^a,c^	*P* value
Biochemically validated abstinence								
6 m	62 (4.8)	26 (6.1)	27 (6.3)	9 (2.1)	0.75 (0.41, 1.38)	0.35	2.67 (1.17, 6.09)	0.02
12 m	72 (5.6)	36 (8.4)	23 (5.4)	13 (3.0)	1.42 (0.79, 2.52)	0.24	2.51 (1.26, 4.99)	0.009
Self-reported 7-day PPA of traditional cigarette								
6 m	153 (11.9)	76 (17.8)	46 (10.7)	31 (7.2)	1.57 (1.03, 2.38)	0.03	2.50 (1.58, 3.97)	< 0.001
12 m	184 (14.3)	108 (25.2)	48 (11.2)	28 (6.5)	2.48 (1.68, 3.65)	< 0.001	4.70 (2.97, 7.45)	< 0.001
Self-reported reduction of ≥ 50% in cigarette consumption ^d^								
6 m	179 (15.8)	63 (17.9)	55 (14.4)	61 (15.4)	1.33 (0.98, 2.01)	0.17	1.17 (0.78, 1.74)	0.45
12 m	202 (18.4)	71 (22.2)	79 (20.8)	52 (13.0)	1.16 (0.79, 1.70)	0.44	1.99 (1.33, 2.98)	0.001
Quit attempts^d^								
6 m	447 (39.5)	221 (51.6)	116 (27.1)	110 (25.7)	2.74 (2.04, 3.68)	< 0.001	3.01 (2.23, 4.07)	< 0.001
12 m	483 (43.9)	271 (63.3)	102 (23.8)	110 (25.7)	6.04 (4.40, 8.30)	< 0.001	5.39 (3.94, 7.37)	< 0.001
Decreasing nicotine dependency by the FTND^d^								
6 m	168 (14.9)	120 (28.0)	25 (5.8)	23 (5.4)	6.93 (4.26, 11.28)	< 0.001	8.77 (5.28, 14.56)	< 0.001
12 m	219 (19.9)	157 (36.7)	30 (7.0)	32 (7.5)	9.99 (6.24, 15.99)	< 0.001	9.68 (6.15, 15.24)	< 0.001

*Referral *participants in the current study receiving the brief advice + active referral, *Self-Quit *participants in the previous RCT receiving brief advice and self-determining to quit immediately or progressively, *Control *participants in the previous RCT receiving a smoking cessation leaflet and placebo treatment

^a^ Estimates from the multiple logistic/linear regression adjusted for age, gender, education level, marital status, employment, cigarette consumption per day, regular tobacco use time, nicotine dependence level by FTND, stage of readiness to quit, quit attempt, referral history, other tobacco use, psychological perspectives on importance, confidence, and difficulty of quitting smoking at baseline

^b^ Reference group refers to the self-quit group

^c^ Reference group refers to the control group

^d^ Quitters were excluded

Table 3 shows that the self-reported 7-day PPA from traditional cigarettes at 6-month (aOR, 1.57; 95% CI, 1.03–2.38; *P* = 0.03) and 12-month (aOR, 2.48; 95% CI, 1.68–3.65; *P* < 0.001), 24-h quit attempt at 6-month (aOR, 2.74; 95% CI, 2.04–3.68; *P* < 0.001) and 12-month (aOR, 6.04; 95% CI, 4.40–8.30; *P* < 0.001), and decreased nicotine dependency levels at 6-month (aOR, 6.93; 95% CI, 4.26–11.28; *P* < 0.001) and 12-month (aOR, 9.99; 95% CI, 6.24–15.99; *P* < 0.001) in the referred group were significantly higher than the participants in our previous RCT who received brief advice and self-determination theory-based interventions.

### Implementation outcomes

Eight sessions were conducted successfully in the EDs at the four hospitals included in the study. Ninety-three HCPs from these participating EDs completed the training workshop, while 76 HCPs referred participants to smoking cessation services. After the evaluation of outcome implementation, the results showed that this study adequately covered the representative institutions and participants (see Table 4 and Additional file 11: Table S7).

**Table 4:** Implementation outcomes of the real-world efficacy for brief advice plus active referrals

**Outcomes/** indicators^a^		**Measures**	
**Reach:** Number, ratios, and representation of smokers meeting inclusion and exclusion criteria in the implementation research project			
Number		1601	
Ratio		61.4%, 1601/2609	
Representation		No significant difference in demographics and smoking profiles between the participants recruited at baseline and participants who completed the whole study at the endpoint (Additional file 11)	
**Efficacy:** Intervention effects at the individual level within the project			
Abstinence outcomes	The efficacy outcomes were presented in Tables 2 and 3 and Additional files 9 and 10		
**Adoption:** Number, percentage, and representation of participating institutions and HCPs in the project			
Number		4 EDs agreed to participate	
Ratio		80%, 4/5	
Representation		We sent invitation letters to 5 of 16 EDs in hospitals throughout different districts of Hong Kong under the Hospital Authority. No significant difference in settings between participating institutions and all institutions meeting the recruitment criteria (Additional file 12)	
Number		76	
Ratio		81.7%, 76/93	
Representation		No significant difference in demographic information between the HCPs participating in the project at baseline and the HCPs who completed the whole study at the endpoint (Additional file 13)	
**Implementation:** Degree of adherence to the intervention, local adjustments made during guideline implementation, and implementation costs			
Fidelity		8 training sessions were conducted in the institutions	
Fidelity		HCPs’ knowledge of the risk of smoking, attitudes towards smoking, tobacco control, and smoking cessation; and self-efficacy to deliver brief smoking cessation advice and active referral were significantly promoted immediately after training but decreased to prior levels within 6 months after the completion of projects compared to pre-training. The trained HCPs were satisfied with the overall content and setting of the training workshop (Additional file 14)	
Implementation costs		The total operating cost of interventions included (1) the personnel for healthcare professional training with 1,079.12 USD (HK$ 8400), (2) The cost of smoking cessation services for the referred participants is 48,690.92 USD (HK$ 379,015), and (3) intervention delivery and equipment with 13,225.06 USD (HK$ 102,945.25)	
**Maintenance:** Maintenance of the project after the research is completed			
Normalization		4	
Fidelity		748	

^a^The definition of each indicator was presented in Additional file 5

Regarding the adoption, we sent invitation letters to 5 of 16 EDs in hospitals throughout different districts of Hong Kong under the Hospital Authority; 4 EDs agreed to participate. No significant difference in settings between participating institutions and all institutions meeting the recruitment criteria (see Additional file 12: Table S8).

A total of 76 HCPs completed the whole study at the endpoint. No significant difference in demographic information between the HCPs participating in the project at baseline and the HCPs who completed the whole study at the endpoint (see Additional file 13: Table S9).

There were significant immediate improvements in the HCPs’ knowledge about smoking risks (from 9.4 to 10.8, *P* < 0.001), attitudes toward smoking control policies (from 9.1 to 9.8, *P* < 0.001), and self-efficacy in delivering brief cessation advice and active referral (from 16.6 to 17.3, *P* = 0.034) at the post-training assessment. However, a 6-month follow-up indicated that these indicators were not sustained and reverted to levels statistically similar to baseline. Furthermore, while post-training satisfaction was high, the perceived long-term impact on HCPs’ practical skills, such as confidence in offering advice, showed a significant decline, suggesting a potential need for booster sessions for HCPs to maintain the effectiveness of the intervention over time (see Additional file 14: Table S10).

The cost-effectiveness analysis demonstrated an ICER of 39,771.25 USD per quality-adjusted life year, which falls within the World Health Organization’s recommended threshold of 1–3 times the gross domestic product per capita in Hong Kong. This finding indicates that active referral is cost-effective (see Additional file 15: Cost-effectiveness analysis) [49–53].

## Discussion

In this large, real-world setting, we found that brief cessation advice combined with active referral was feasible to implement as part of usual care in ED settings and may contribute to clinically meaningful increases in smoking abstinence. Despite the low rate of biochemically validated abstinence following a high refusal rate for validation visits due to the COVID-19 pandemic, the participants self-reported their increased abstinence and reduced cigarette consumption. Moreover, the intervention also effectively improved their cessation intentions and attempts. The present findings underscore the potential of referral strategies in bolstering smoking cessation efforts and fostering a proactive approach to quitting among smokers.

Compared with no referral, active referral interventions were associated with notable benefits in smoking cessation outcomes. Specifically, the referred group showed higher rates of biochemically validated abstinence, 7-day self-reported cigarette abstinence, reduced cigarette consumption, and increased readiness to quit and quit attempts at both the 6- and 12-month follow-ups. Moreover, the referred participants also had significantly better nicotine dependency levels and more quit attempts compared with those who received only brief cessation advice in our previous trial. The non-RCT design of this study and potential residual confounding factors make it challenging to establish a direct causal relationship between referrals and smoking cessation outcomes. Nevertheless, these findings suggest that active referral may enhance the effectiveness of ED-based smoking cessation interventions. The PSM results revealed the robustness of referral interventions in aiding smoking cessation and highlighted their potential to reduce smoking behavior and consumption over time.

Pope et al. [54] observed low engagement rates with smoking cessation services in ED settings, which were primarily due to barriers such as participants not being contacted by smoking cessation services and the offered support not meeting their needs. Our study encountered similar barriers in the referral process. Post-discharge engagement was further hindered by missed calls, scheduling difficulties, and resource constraints. Despite these challenges, various strategies, such as participant education, enhanced communication, and streamlined protocols, have demonstrated the potential to overcome these barriers and sustain smoking cessation interventions in routine clinical practice. This proactive approach likely contributed to the higher engagement and smoking cessation rates observed in our study. Future research could further examine how active referral mechanisms can be adapted to different health-care settings to address similar challenges.

The findings that pronounced disparities across various cessation metrics among the referred group versus the unreferred group and their quit rates were similar to those of the self-quit group in our previous trial. These findings shed light on the importance of HCPs’ brief cessation advice. The main goal of the brief advice intervention was to initiate a positive change in smoking behavior. At the baseline, 88.9% of the participants were in the pre-contemplation stage, indicating no intention to quit smoking. Following the intervention, the participants demonstrated significant improvements in readiness to quit, as shown by their progression through the transtheoretical model (TTM) stages. This was especially evident among those successfully referred to smoking cessation services, whose intentions showed greater progression through the TTM stages. The AWARD model of brief advice was effective in motivating individuals initially ambivalent to seriously consider quitting and to take further action by discussing the harmful effects of smoking and the benefits of quitting [55]. Although brief advice is concise, it can still be highly personalized. Therefore, HCPs can tailor their recommendations based on smokers’ individual circumstances, including their health status, smoking history, and readiness to quit [56]. Addressing these factors increases the likelihood of successful smoking cessation outcomes.

Regarding the implementation of this research project, as evaluated through the lens of the RE-AIM framework, this study successfully engaged 1601 individual smokers, representing a substantial proportion of the target population, with no significant disparities observed in demographics or smoking profiles between the participants recruited at baseline and those who completed the entire study. Adoption metrics indicate strong institutional engagement, with four out of five eligible organizations participating. Although 40 staff were initially planned to be trained, 93 HCPs ultimately completed the training, and 76 HCPs were involved in the following interventional work referring participants to smoking cessation services due to their high interest and enthusiasm. This highlighted the feasibility and acceptability of implementing such interventions among HCPs as routine strategies in their clinical ED settings. In terms of implementation, fidelity to the intervention was demonstrated through the delivery of half-day training sessions and enhanced knowledge and satisfaction among HCPs. Training HCPs working in EDs in the required knowledge, attitudes, and practices related to smoking cessation may enhance the capacity of these clinics and promote healthy communities in the long run. Training HCPs to deliver brief advice, actively refer smokers, and equip them with the necessary skills to continuously promote smoking cessation are cost-effective strategies [57]. In the present study, we promoted abstinence rates without using products containing nicotine (e.g., NRT or ECs with nicotine) in efforts to stop smoking and achieve cost-effective and complete cessation. Most importantly, this study initiated the establishment of a network of HCPs, clinics, and non-governmental organizations that could eventually provide effective smoking cessation services to smokers in both clinical and community settings.

Future implementations should address the challenges and suggestions of this study. First, the transient nature of encounters with smokers during ED visits limits the time available for HCPs to deliver comprehensive interventions. Balancing the provision of brief advice with ensuring an effective referral process requires streamlined protocols and well-established partnerships with smoking cessation services [48]. Additionally, smokers’ willingness and readiness to quit may vary during ED visits, necessitating tailored motivational strategies and follow-up support to increase their engagement and adherence to recommended interventions. Integrating brief smoking cessation advice and active referrals into ED settings is crucial for HCPs, allowing them to address smoking proactively and help reduce tobacco-related diseases by incorporating standardized protocols into routine ED practices [56]. Collaboration between EDs and community-based smoking cessation services is essential for seamless post-discharge patient care. Additionally, EDs can play a crucial role in advocating for policy changes, allocating resources to cessation services, promoting smoke-free environments, and implementing systems to monitor patient outcomes.

### Limitations

This study is limited by its non-randomized trial design; however, we focused on evaluating the intervention’s success in promoting smoking cessation in real-world settings. A single-group pre- and post-test design was used in which regression using PSM with adjustments may help to provide evidence for the effectiveness of the intervention. We acknowledge that performing a power calculation in a single-arm, non-controlled study has inherent limitations. The sample size estimation was based on assumptions drawn from our previous RCT, and the comparison between the referred and unreferred groups in this study did not equate to a formal control group. Nonetheless, the results of the power calculations offered a practical framework to guide the study design and ensure sufficient statistical power to detect differences in the primary outcome. Second, during the COVID-19 pandemic, many participating HCPs could not conduct follow-up calls due to their busy workloads. In addition, a large number of patients who reported that they had quit smoking refused to participate in biochemical validation due to their concerns about potential infection risks, leading to low participation in biochemical validation. Future studies should evaluate the behavior of HCPs and patients when they are not affected by the COVID-19 pandemic. Considering the participants, self-reported outcomes were more reliable than biochemically validated outcomes in this case. Third, in the current real-world setting, booster follow-up calls were not routinely conducted by ED nursing staff or other HCPs. This deviation from standard practice may limit the generalizability and direct applicability of the study’s findings to clinical settings. Nevertheless, the study results provide valuable insights that could inform future clinical practice, such as encouraging the integration of follow-up calls as a supportive strategy to assist smokers in quitting. The booster follow-up calls, administered exclusively to participants who accepted the referral, may partly or entirely account for the differences observed in smoking abstinence rates. Future trials should consider the potential impact of such follow-up support when evaluating the effectiveness of referral-based interventions. Additionally, while a cost-effectiveness analysis was conducted, some of the modeling data were derived from previously published studies rather than collected directly in this study, which may have affected the precision of the analysis. Future economic evaluations could provide more robust evidence to support the implementation of this intervention.

## Conclusions

This trial demonstrated that brief smoking cessation advice and active referral can be feasibly integrated into usual care for patients who smoke in ED settings. This innovative and cost-effective approach could motivate HCPs to assist more smokers with smoking cessation in routine clinical settings and improve smoking abstinence levels. After optimizing the training and implementation routines, the intervention can be further implemented and generalized into both clinical and community settings.

## Supplementary Information

Additional file 1. Protocol. An evidence-based smoking cessation intervention comprising brief advice plus active referrals for smokers attending emergency departments: protocol for a REAIM framework guided implementation study.

Additional file 2. TREND Statement Checklist.

Additional file 3. Questionnaires-Healthcare Professionals. Baseline, post training, and 3/6-month follow-up Questionnaires.

Additional file 4. Questionnaires-Patients. Baseline and 6/12-month follow-up Questionnaires.

Additional file 5. Table S1. RE-AIM Evaluation indicators.

Additional file 6. Table S2. Schedule of assessments.

Additional file 7. Table S3. Comparison of baseline characteristics and smoking profiles between those who were referred to smoking cessation services in the original unmatched sample and the propensity-score matched sample.

Additional file 8. Table S4. Smokers’ demographic and smoking profile at baseline and difference among referral, self-determination and control group in the original unmatched sample.

Additional file 9. Table S5. Smokers’ demographic and smoking profile at baseline and difference among referral, self-determination and control group in the propensity-score matched sample.

Additional file 10. Table S6. Sensitivity analysis using GEE, multiple imputed outcomes, and completed cases.

Additional file 11. Table S7. Similarity of demographics and smoking profiles between the participants recruited at baseline and participants completed the whole study at the endpoint.

Additional file 12. Table S8. Similarity between participating institutions and all institutions meeting the recruitment criteria.

Additional file 13. Table S9. Similarity of demographics information between the HCPs participating in the project at baseline and the HCPs completed the whole study at the endpoint.

Additional file 14. Table S10. Healthcare professionals’ knowledge about the risks of smoking, attitudes towards smoking, self-efficacy to deliver brief smoking cessation advice and active referral, and satisfaction with and feedback on the training workshop at pre-training, post-training, 6-month follow-ups after the research completed.

Additional file 15. Cost effectiveness analysis.

## Data Availability

The datasets used and/or analysed during the current study are available from the corresponding author on reasonable request.

## Abbreviations

Eds: Emergency departments
AWARD: Ask, Warn, Advise, Refer, Do-it-again
HCPs: Health care professionals
RCT: Randomized controlled trial
PPA: Point prevalence abstinence
ppm: Parts per million
HKD: Hong Kong Dollar
US$: United States Dollar
RE-AIM: Reach, Effectiveness, Adoption, Implementation, Maintenance
TREND: Transparent Reporting of Evaluations with Nonrandomized Designs reporting guideline
ITT: Intention-to-treat
OR: Odds ratio
CI: Confidentiality interval
GEE: Generalized estimating equation
AR1: AutoRegressive Order 1
PSM: Propensity score matched
ICER: Incremental cost-effectiveness ratio
