# Supplementary material for: Evidence-based brief cessation advice plus active referral for emergency department patients who smoke: a single-arm, real-world clinical trial
Source: BMC Med. 2025 Nov 27;23:714. doi: 10.1186/s12916-025-04534-9 (PMC12751522; doi:10.1186/s12916-025-04534-9)
Supplement: Supplementary file 1 — Additional file 1. Protocol. An evidence-based smoking cessation intervention comprising brief advice plus active referrals for smokers attending emergency departments: protocol for a REAIM framework guided implementation study. [file 12916_2025_4534_MOESM1_ESM.pdf]

Protocol

An evidence-based smoking cessation intervention comprising brief advice plus active referrals for smokers attending emergency departments: protocol for a RE-AIM framework guided implementation study

Introduction

Smoking exerts harmful effects on nearly every organ of the body and is considered responsible for 8 million deaths worldwide every year.[1] Despite a decrease in the prevalence of daily cigarette smoking from 23.3% in 1982 to 10.2% in 2019, 652,000 daily smokers[2] remain in Hong Kong, where 400,000 hospitalisations per year are attributable to smoking.[3]

Previous studies indicated that healthcare professionals play a crucial role in facilitating and promoting smoking cessation as their provision of smoking cessation counselling can more effectively increase cessation rates.[4,5] Visiting emergency departments (EDs) presents an excellent teachable moment for encouraging smoking cessation as smokers who consult physicians in an emergency setting had more motivations to quit under current situation, therefor, they may be more likely to adopt better health-related behaviours.[6] In addition, according to the Hospital Authority, approximately 68% of 2 million people visit EDs in Hong Kong each year are triaged as semi-urgent or non-urgent.[7] The average waiting time for a medical consultation generally exceeds 30 mins and 1–2 h, respectively, which represent a golden opportunity to deliver available smoking cessation programmes.[7]

Most existing cessation programmes, including stage-matched interventions and motivational interviews, generally require implementation periods exceeding 30 minutes.[8,9] Previous reviews have indicated that smoking cessation strategies at EDs could effectively increase the cessation rate.[10,11] In addition, compared to brief advice, a comprehensive intervention might be more effective in promoting smoking cessation.. [12-14] However, the provision of a comprehensive smoking cessation intervention is not feasible in the busy clinical settings. [15] Other barriers to assistance include a lack of training and experience, lack of confidence in the efficacy of the interventions and deficiencies in or a lack of incentives, support or requirements from hospital management regarding the implementation of these programmes.[16,17] Moreover, our previous smoking cessation projects in an outpatient clinical setting revealed that many

patients were reluctant to participate because of impatience to a long intervention or concerns of missing or delays in their medical procedures.[15,18,19] A brief intervention would be cost-effective and more feasible for routine use in clinical practice by all or most healthcare professionals after minimal training. Therefore, an innovative intervention is needed and should be evaluated with the intent to enhance its effectiveness and potential for implementation in clinical settings.

Cigarette smoking is addictive, and cessation is a difficult process associated with a high rate of relapse, particularly among smokers with a high level of nicotine dependency.[20] According to the latest Hong Kong Thematic Household Survey, 31.2% of participating cigarette smokers had tried but failed to quit smoking.[2] Although a brief intervention is more feasible in a clinical setting, it may be too brief and inadequate to have a long-term effect on smokers.[18,19] Moreover, the effectiveness of some brief interventions for smokers which focus mainly on providing self-help materials is undermined by a failure to offer or arrange any follow-up.[12] Evidence indicates that smoking cessation services, including telephone quitlines, are an effective means of supporting smoking cessation efforts.[21] However, these services are generally poorly utilised, with a usage rate of 23.2% among smokers in Hong Kong.[2] Therefore, the combination of brief cessation advice plus referrals for smokers to utilise existing smoking cessation services in Hong Kong may comprise an alternative strategy that could enhance the effects of intervention, particularly for patients requiring additional counselling.

We conducted a previous RCT in EDs using an AWARD (Ask, Warn, Advise, Refer and Do-it-again) model-based brief smoking cessation intervention, in which we allowed smokers to select their own quitting schedules (e.g., immediately or a progressive reduction in the number of cigarettes smoked with the ultimate goal of complete cessation over an acceptable period).[22] The results showed that it could effectively promote smoking cessation among people attending emergency departments. In addition, the subjects were more willing to adhere to their own schedule as a result of an increase in autonomy.[23] This study demonstrated that such brief smoking cessation intervention could be used feasibly in emergency departments and was accepted by both healthcare professionals and smokers.[20] On the other hand, The active referral of smokers to existing smoking cessation services where they can receive more comprehensive counselling has been proven to effectively improve the rates of cessation. [24-26] Based on the health needs of the community, we propose that such a smoking cessation strategy should combine different components, such as the administration of brief advice by healthcare professionals and the

provision of referrals and follow-up boosters by community smoking cessation services, to achieve a larger intervention effect.

As stated above, a brief intervention would be cost-effective and more feasible for routine use in clinical practice by all or most healthcare professionals in a hectic acute care setting after minimal training. The combination of smoking cessation intervention plus an active referral showed that an intervention involving brief advice, early proactive contact, and referral to smoking cessation service providers enhanced the use of these services and significantly increased abstinence among ED smokers who participated in our prior RCTs.[24-27] Nevertheless, how to translate the integrated intervention into daily clinical practice and how it can be performed in the real-world situation remain uncertain. A standard evaluation of the implementation effect should be further conducted to guide the practice. Over the past decades, the study team has established good collaborative relationships with local smoking cessation service providers, whereby the results of effectiveness trials can be directly translated into policy mandates and best practice guideline. The overarching goal of this study is to address the research to practice gap and implementation of the intervention. The specific objectives are (1) to promote this evidence-based project to emergency departments in various hospitals under the Hospital Authority; (2) to construct a network with non-governmental organisations (NGOs) to provide smoking cessation services; (3) to train healthcare professionals to use the AWARD model to deliver brief cessation advice to smokers; (4) to deliver brief cessation advice via healthcare professionals and actively refer smokers to existing smoking cessation services; and (5) to evaluate programme implementation outcomes.

<u>Implementation science and conceptual frameworks</u>

Recent commentary has noted conceptual frameworks that can potentially inform the design of investigations that target the widespread dissemination and implementation of health care interventions.[28] The reach effectiveness adoption implementation maintenance (RE-AIM) evaluation framework underlying the design and implementation is used to better understand the potential uptake of the intervention implemented in this study.

RE-AIM has been used to plan health interventions, evaluate health interventions, evaluate health policy impact, assess the literature, and to compute composite metrics to estimate intervention impact.[29] It outlines clear stages of assessment for both effectiveness and implementation outcomes, and provides a model for the integration of pragmatic trial results into routine clinical practice, which comprised of five indicators: Reach, Efficacy, Adoption, Implementation and Maintenance (**Table 1**).[30] Reach is defined as

the number or percentage of the population and the representativeness of those included in the program or study. Efficacy measures change in the outcome variables of interest, including impact on quality of life and any adverse effects on intervention. Adoption measures the proportion and representativeness of staff and settings that adopt a given program. Implementation assesses the extent to which a program or policy is delivered consistently, and the time and costs of the program. Maintenance assesses the long-term effects and attrition in the project, both of individuals and organizations. This includes the extent of discontinuation, modification, or sustainability of program.[31] With the advantages of being contextual, practical and having robust evidence of its applicability across a wide array of interventions, populations, settings and health behaviors, RE-AIM offers a systematic framework for expanding beyond the usual measures of effectiveness, to the broader criteria of internal and external validity and attends to the characteristics of programs and interventions that ensure these can be readily adopted, widely implemented, and sustained.

Table 1. RE-AIM Evaluation dimensions

Dimension	Level
Reach (proportion of the target population that participated in the intervention)	Individual
Adoption (proportion of settings, practices, organization and plans that will adopt this intervention)	Organization
Efficacy (success rate if implemented as in individual guidelines; defined as positive outcomes minus negative outcomes)	Individual
Implementation (extent to which the intervention is organization implemented as intended in the real world)	Organization
Maintenance (extent to which a program is individual, and organization sustained overtime)	Individual and organization

Methods and analysis

Design overview and target population

To address the research to practice gap and implementation of an evidence-based smoking cessation intervention comprising the provision of brief cessation advice and active referral to existing smoking cessation services to smoking at the EDs, we will conduct a three-phase study, including 1) project promotion, 2) recruitment and training, and 3) project implementation. The efficacy of the intervention has been evaluated in a previous RCT.[22] To maximum the accessed population and minimum the overall cost, a single-group pre- and post-test design will be used to evaluate the overall implementation effectiveness of the intervention in promoting smoking cessation in real-world clinical practice.

Participants in this study included both healthcare professionals and patients in EDs. Healthcare professionals who are employed in the emergency departments of hospitals under the Hospital Authority in Hong Kong will be invited to participate in this study and trained to deliver the intervention. Chinese smokers who present at the selected EDs and fulfilled the inclusion criteria will be invited to participate in this study. The inclusion criteria were (1) age 18 years or older, (2) triage as semi-urgent or non-urgent, and (3) current smokers (occasional or daily). Smokers will be excluded if they were unable to give informed written consent or receive service because of impaired mental status, cognitive impairment, or communication barriers or if they has participated in other smoking cessation programs or services.

Project promotion

Prior to the initiation of recruitment and intervention implementation, we will send letters of invitation containing the details of this project and a solicitation for participation to emergency departments in EDs in 18 major acute-care hospitals in different districts of Hong Kong managed by the Hospital Authority.[32]. To ensure the representative of the findings, all EDs will be invited with at least 5 hospitals from different districts will be enrolled in this study. We anticipate that 5–8 emergency departments at various hospitals will agree to participate. The project team will organise meetings with the Chiefs of Services (COSs) and Department Operation Managers (DOMs) to explain the project and address their concerns. The COSs or DOMs of emergency departments that agree to join the project should encourage and nominate staff members (i.e., healthcare professionals) to attend a half-day training session organised by the project team.

Healthcare Professional Training

Based on our experience in previous studies, about 10 trained healthcare professionals will be required in each EDs to ensure the continued implementation of the intervention within a week. Given that, approximately 50–80 healthcare professionals from participating emergency departments will be recruited to attend a half-day training workshop. The training content will mainly address smoking and health, smoking cessation services and tobacco control policies in Hong Kong. Additionally, the use of a brief smoking cessation intervention based on the AWARD model to advise smokers to quit immediately or quit progressively, with the ultimate goal of completing cessation over an acceptable period, and the procedures for referring smokers to existing smoking cessation services will be discussed. The trained healthcare professionals will be encouraged to deliver brief smoking cessation advice based on the AWARD model to smokers who attend emergency departments.

Intervention implementation

Smokers who present at the selected emergency departments and triage as semi-urgent or non-urgent will be invited to participate in the study. Smokers will receive a leaflet about the health-related hazards of smoking and benefits of quitting, as well as a pocket-sized information card containing brief information about the existing smoking cessation services in Hong Kong (e.g., smoking cessation telephone hotlines, addresses and operational hours). Then the healthcare professionals will provide brief counseling using AWARD model includes five components and can be delivered within a minute: (1) Ask about smoking

history; (2) Warn about the high risk of smoking; (3) Advise to quit as soon as possible and comply with the decided quit date; (4) Refer smokers to smoking cessation services; and (5) Do it again. In the fourth step, the healthcare professionals will describe the existing smoking cessation services to smokers, and actively refer them to the chosen service provider within 1 week of recruitment. Smokers will provide their consent and contact details (name and telephone number) to the project team. Subsequently, smokers will receive proactive telephone calls from the smoking cessation service providers for cessation counselling or booking an appointment at the smoking cessation clinic. Booster follow-up calls at 1 week and 1 month after baseline will be conducted to enhance and confirm the referral of the patient participants without the evaluation of outcomes.

Assessment

Following the conceptual frameworks, the assessment includes the primary effectiveness evaluations, which are patients-reported outcome measures, and implementation outcome evaluations informed by the RE-AIM evaluation framework.

A. Effectiveness evaluation

Following the recommendations by the Society of Research on Nicotine and Tobacco (SRNT),[31] the primary outcome is the biochemically validated abstinence at 6 months. The self-reported quitters at the 6-month follow-up will be invited to participate in a biochemical validation (the measurement of exhaled [CO] and salivary cotinine level). The criteria for validated abstinence are an exhaled CO level of less than 4 ppm and a saliva cotinine level of less than 10 ng/ml. Those who pass both tests will be regarded as a biochemically validated quitter, while those who absent or fail to the biochemical validation test will be regarded as current smokers.[33]

The secondary outcomes are (1) biochemically validated abstinence at 12 months, (2) self-reported 7-day point prevalence of abstinence at 6 and 12 months, (3) self-reported reduction of ≥ 50% in cigarette consumption at 6 and 12 months.

B. Implementation evaluations

Adoption: The ratio of the number of EDs participating in this study and the total number of EDs in Hong Kong will be used to assess the adoption of the programme.

Reach: The ratios of the number of participants actually served and the total number of participants ideally served for both healthcare professionals and patient participants will be used to assess the "reach" index of the programme. Healthcare professional and patient participants ideally served are those who meet the criteria for this study in Hong Kong during the study period. The number will be estimated according to the data from the Hospital Authority.

Efficacy: Prior to the design of the study, we conducted both quantitative and qualitative studies to identify the needs of new intervention for smokers in the hospitals. According to the need of target smokers, we have conducted randomized controlled trials identify that both the brief cessation advice and active referral to existing smoking cessation services were effective in promoting the smoking cessation among smoking patients at the EDs in Hong Kong. In this programme, anticipated consequences including the patients-reported outcomes, the number of participants referred to smoking cessation services, and the consistency of intervention, including the intended and the time and cost of delivering the intervention among clinicians and centers among the target population; benefits, such as the promotion of their health condition, especially the illness leading them to visit the EDs, money saved, time of hospital visiting reduction; and adverse consequences, such as weight gain will be recorded according to the feedback from the healthcare professionals and patient participants to further assess the effectiveness of the intervention in actual implementation.

Implementation: The improvements of (1) the knowledge of the risk of smoking; (2) attitudes towards smoking, tobacco control and smoking cessation; (3) the self-efficacy; (4) intention, and (5) planning to deliver smoking cessation advice; (6) the practices of delivering smoking cessation advice; and (7) the actual number of delivering brief advice and referral among healthcare professionals will be used to assess the implementation of the programme.

Maintenance: Both long-term follow-ups for patients and healthcare professionals will be used to assess the maintenance of the programme. In addition, the feedback from patients and healthcare professionals on the barriers and facilitators to receiving/applying the interventions will be considered to prolong the maintenance of the implementation. The feedback interview will be conducted at 12 months follow-up with a guide. The operating cost of interventions, including the personnel for healthcare professional training, healthcare professional and patient-participants recruitment and intervention delivery and equipment (e.g., print-based materials and referral cost), will be calculated and provided.

Data collection

As shown in figure 1, at the project promotion phase, the number of emergency departments that agree to join the projects will be recorded.

At the healthcare professional training phase, we will record the number of healthcare professionals who register for the training workshops. A standardised questionnaire will be administrated to the healthcare professionals. The healthcare professional participants will complete a pre-test before the training workshop (T1) and a post-test afterward (T2), which will also ask for contact information for further follow-up, assess the healthcare professional participants' responses on whether the training workshop met their learning needs and expectations and request suggestions for future improvement of the training workshop. An experienced research nurse who delivers the training and a senior research assistant who has been working on the clinical smoking cessation project for several years will be recruited in this study as external facilitation, including visiting the EDs enrolled in our study at least once per week to answer the question and problems in implementation of the proposed intervention, recording all questions and giving feedback in next visit.[34] At 3 (T3) and 6 months (T4) after the training workshop, the trained healthcare professionals will be followed up by telephone using the standardised questionnaire to assess the long-term effects of the training workshop, including the case number referred by them in the past period, self-efficacy, difficulties, and barriers on implementation the intervention. A brief feedback interview will be conducted by the research nurse to the healthcare professionals according to their response contents of the questionnaire.

At the intervention implementation phase, we will evaluate the number of smokers that have been approached by healthcare professionals; the number of smokers that have been given brief advice on smoking cessation; and the number of smokers that have been referred to smoking cessation services. A structured questionnaire validated in our previous study will be used to collect the patient-participants' demographic, smoking characteristics, and previous exposure of usual smoking cessation services at baseline.[18,19,22] Booster follow-up calls at 1 week and 1 month after baseline will be conducted to enhance and confirm the referral of the patient-participants without the evaluation of outcomes. Follow-ups will conducted at 6 months and 12 months using the structured questionnaire. The self-reported quitters at the 6 and 12 months follow-up will be invited to participate in a biochemical validation. A brief feedback interview will be conducted to the patient-participants according to their abstinence and referral results. The schedule of the assessments was presented in **Table 2**.

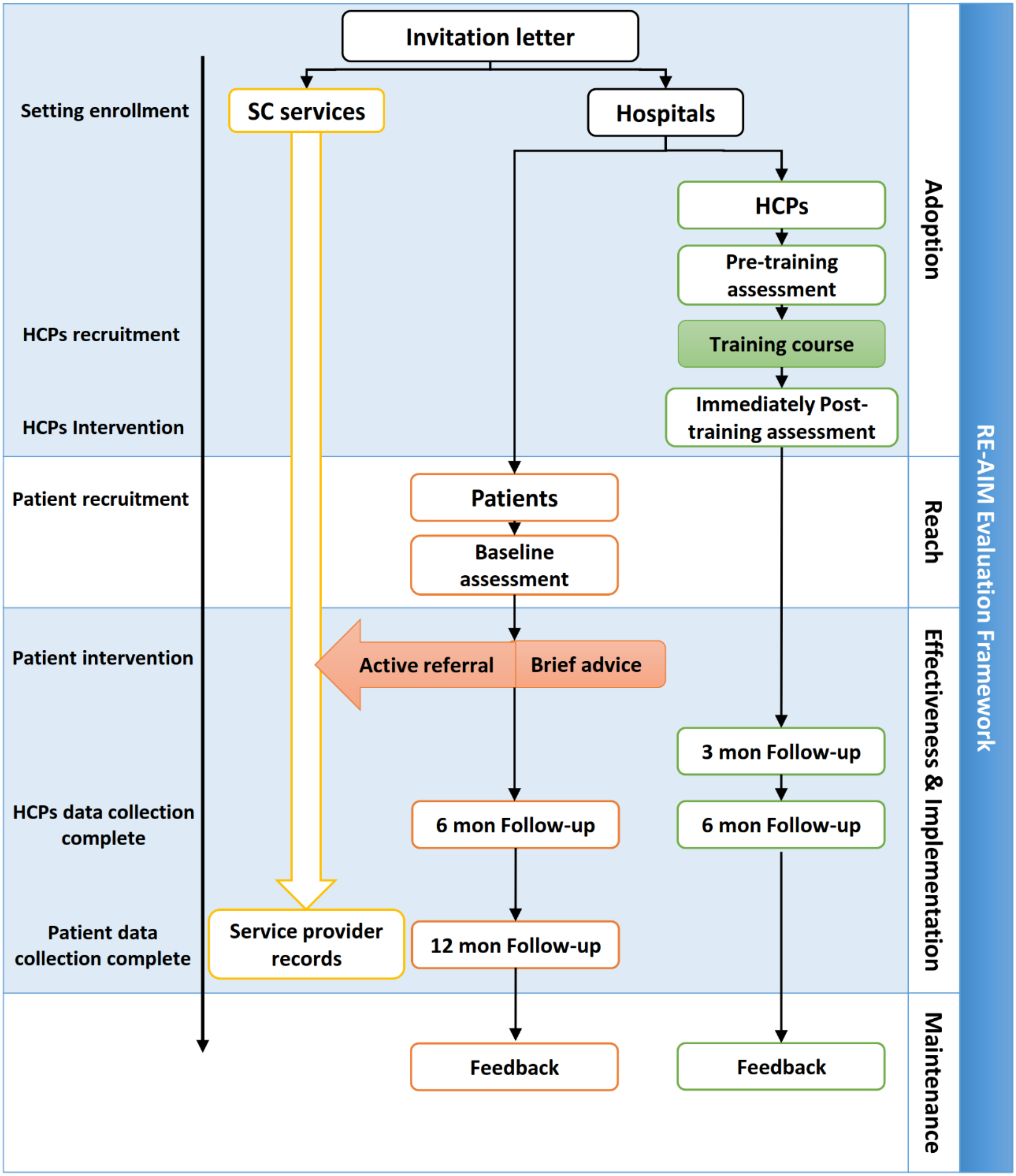

Figure 1. The programme procedure flow

Table 2. Schedule of assessments

Assessment	Time-point					
	Baseline	1 week	1 month	3 Months	6 Months	12 Months
Informed consent for healthcare professionals (HCPs)	×					
Informed consent for participants	×					
Eligibility screen for participants	×					
Intervention initiation	×					
Sociodemographic characteristics *	×					
Intervention booster calls		×	×			
Effectiveness evaluation						
Biochemically validated abstinence					×	×
Self-reported 7-day point prevalence of abstinence					×	×
Self-reported reduction of cigarette consumption					×	×
Implementation evaluations						
Number of EDs participating in this study	×					
Participants enrolled in this study	×					
Number of participants referred to smoking cessation services	×	×	×		×	×
HCPs' knowledge of the risk of smoking	×			×	×	
HCPs' attitudes towards smoking, tobacco control and smoking cessation	×			×	×	
HCPs' self-efficacy to deliver smoking cessation advice	×			×	×	
HCPs' intention to deliver smoking cessation advice	×			×	×	
HCPs' planning to deliver smoking cessation advice	×			×	×	
HCPs' practices of delivering smoking cessation advice	×			×	×	
Number of delivering brief advice and referral among healthcare professionals	×			×	×	
Feedback Interview for HCPs				×	×	
Feedback Interview for participants					×	×

* Sociodemographic characteristics include age, sex, marital status, education level, and occupation.

The justice will be served, as do ensure healthcare professional and patient participants' privacy by maintaining confidential and anonymous records of data collected. All raw data and study documents will be kept in a safe place for five years. The researcher is responsible for its safekeeping and the only one who would have access to the raw data or study record during or after the study. After the period of storage, all raw data and records will be destroyed.

Patient and Public Involvement

No patient involved.

Statistical analysis plan

Due to the sample cohort of smokers will be followed longitudinally before and after the intervention in this study, the sample size is estimated based on paired proportions.. According to our previous RCT involving emergency departments, the smokers had a biochemically validated quit rate of 6.8% in the intervention group when compared with 2.7% in the control group at 6 months.[22] Assuming that 2.7% of the patient-participants could quit smoking before accepting the intervention at baseline and 6.8% of the patient-participants could quit smoking at 6 months after accepting the intervention. Considering a cluster size of 5, to achieve a power of 80% and a two sided significance level of 5%, the study would require a sample size of 1098 pairs.[35] According to our experience, the retention rate at 12 months follow-up is about 70%, given that, at least 1569 smokers will be provided brief smoking cessation advice by healthcare professionals.

The Statistical Package for Social Sciences (SPSS: Version 25; SPSS Inc., Chicago, IL, USA) for Windows will be used to perform the data analysis. The intention-to-treat (ITT) method will be adopted to impute the smoking outcomes in which the cases lost to follow-up will be regarded as smokers. Missing values of demographic characteristics at baseline will be handled by using multiple imputation.

Descriptive statistics will be used to describe the number of emergency departments agree to join the projects as well as the number of smokers advised by the healthcare professionals and referred to receive more smoking cessation counselling provided by the

existing smoking cessation services, their self-reported 7-day point prevalence quit rate, rates of smoking reduction by 50% and quit attempt, and biochemically validated quit rate, as well as the implementation outcomes.

Considering the advantage in taking into account the correlation of the repeated measures within a person, Generalized Estimating Equations (GEE) will be used to assess predictors for the change in the participants' self-reported 7-day point prevalence quit rate, rates of smoking reduction by 50% and quit attempt, and biochemically validated quit rate and the changes in smoking cessation-related knowledge, attitudes, and practices among the healthcare professionals. Subgroup analysis will be further conducted to explore whether the intervention will be performed consistently between participants with different characteristics, including sex, education level, income, and occupations. It is a powerful and versatile procedure for analyzing longitudinal data under minimal assumptions about time dependence and allowed us to use all available longitudinal data, regardless of single missing values at follow-up.[36]. Characteristics of the patient-participants who are successfully referred and not will be compared to explore any modification factors that potentially affect the healthcare professional and patient-participants' adoption of the intervention.

In addition, a thematic analysis framework will be used to integrate the findings from the key information feedback with the implementation results.[37] The data analysis process will begin immediately after each individual interview using NVivo version 12 (QSR International Pty Ltd, 2018). Codes, categories and themes generated will be compared with the established taxonomy for evaluating intervention quality related to behavioural change technique for smoking cessation.[38] Results of the mixed-method analysis will be presented through a number of modalities that may include key information narratives, tabular representation of themes with illustrative quotes, and thematic counts.

Potential limitation

Two caveats should be considered when the findings of this proposed study are interpreted. First, this study did not adopt a design of RCT, which may weaken the association of smoking cessation outcomes among the patient participants and the proposed

intervention. However, as stated above, the efficacy of the intervention has been evaluated in previous RCT. Current study mainly focuses on evaluate the implementation outcomes of the intervention in promoting smoking cessation in real-world settings. A design of single-group pre- and post-test design is therefore recommended. Second, any implementation study will not guarantee ‘full’ intervention compliance by all providers and recipients. For instance, the healthcare professional participants in this study will be encouraged to apply the interventions to all eligible patient participants they accessed. But they will possibly omit this procedure during the extremely busy situation or refused by the patients. In addition, the patient participants are voluntary to be referred to the smoking cessation services. Thus, they will be able to choose to either be referred or refuse the referral, and either visit the referred smoking cessation services or refuse to be actively accessed and visit the services throughout the intervention period. As a result, although the study will show the effectiveness of referral, we expect that there will be a variety of levels of participation. Nevertheless, the levels of participation will be archived for further content and compliance analyses. The actual effect of participating adhering to the proposed intervention will be investigated by filtering out participants who exhibit low adoption.

Ethics and dissemination

Ethical approval for this study has been obtained from the Institutional Reviewer Board of the University of Hong Kong/Hospital Authority Hong Kong West Cluster (UW19-032). The protocol has been reviewed by the board of the Health and Medical Research Fund (HMRF) administered by the Food and Health Bureau in Hong Kong. This study wans prospectively registered in ClinicalTrials.gov (Identifier NCT03818360). Written informed consent will be obtained from all healthcare professionals and patient-participants in all study sites after the purpose of the study is explained. They will be informed that their participation in the study, including referral/being referred, and follow-up evaluation and feedback interviews are voluntary and they can withdraw from the study at any time without penalty or loss of benefits. Patients or the public were not involved in the design, or conduct, or reporting, or dissemination plans of this study. The findings from this study will be disseminated locally and internationally through manuscript publications

in peer-reviewed journals and conference presentations at national and international platform. We propose an evidence-based smoking cessation intervention that comprises brief cessation advice given by healthcare professionals plus the active referral of smokers to existing smoking cessation services. This protocol outlines the rationale, design, and methods for process and feasibility evaluations of this complex intervention for smokers attending emergency departments, with the guide of the RE-AIM framework. The evaluation is crucial to explain the study results and to determine the feasibility of scaling the intervention in a Hong Kong healthcare context. The data and results will be used to identify and address issues in the intervention and improve the delivery of the model long-term, with a focus on effectiveness, quality and safety, and scalability.

This project will enhance the community's capacity to promote health in the long run by training the healthcare professionals in the emergency departments with knowledge, attitude, and practice on smoking cessation. They will be trained to deliver brief advice plus active referral to smokers attending their emergency departments and equipped with the necessary skills to continuously play an important role in promoting smoking cessation in the long run as the intervention is brief and cheap. Most importantly, this project will build a network involving healthcare professionals and NGOs that will eventually provide effective smoking cessation services to smokers in both clinical and community settings.

In conclusion, outcomes from this study will directly contribute to the implementation priorities of the EDs in the hospital and the smoking cessation services in Hong Kong. This innovative and cost-effective approach will motivate more healthcare professionals to assist smokers with cessation in routine clinical settings and improve smoking abstinence levels and reduce mortality.

Reference

1. World Health Organization. Tobacco fact sheet. Updated May 2020. https://www.who.int/en/news-room/fact-sheets/detail/tobacco. Accessed June 26, 2020.
2. Census & Statistics Department. Pattern of Smoking. Thematic Household Survey Report No. 70: Pattern of Smoking. Hong Kong: Hong Kong Census & Statistics Department; 2020.
3. Chau J, McGhee SM, Lam TH. Economic costs attributable to smoking in Hong Kong in 2011: a possible increase from 1998. Nicotine Tob Res. 2017;1:8.
4. WHO Tobacco Free Initiative (2010) The Role of Health Professionals in Tobacco Control. World Health Organization, 2005. http://www.paho.org/English/AD/SDE/RA/bookletWNTD05.pdf
5. Movsisyan NK, Varduhi P, Arusyak H, Diana P, Armen M, Frances SA. Smoking behavior, attitudes, and cessation counseling among healthcare professionals in Armenia. BMC Public Health. 2012; 12(1):1028.
6. Bernstein SL, Boudreaux ED, Cabral L, et al. Nicotine dependence, motivation to quit, and diagnosis among adult emergency department patients who smoke: a national survey. Nicotine Tob Res. 2008;10(8):1277-1282. doi:10.1080/14622200802239272
7. Hospital Authority. Hospital Authority Statistical Report (2011-2012). http://www.ha.org.hk/upload/publication_15/471.pdf
8. Prochaska JO, DiClemente CC. Transtheoretical Approach: Crossing Traditional Boundaries of Therapy. Dow Jones-Irwin: Homewood, Ill, 1984.
9. Heckman CJ, Egleston BL, Hofmann MT. Efficacy of motivational interviewing for smoking cessation: a systematic review and meta-analysis. Tob Control. 2010;19: 410-6.
10. Katz DA, Vander Weg MW, Holman J, et al. The Emergency Department Action in Smoking Cessation (EDASC) trial: impact on delivery of smoking cessation counseling. Acad Emerg Med. 2012;19: 409–20. https://doi.org/10.1111/j.1553-2712.2012.01331.x
11. Pelletier JH, Strout TD, Baumann MR. A systematic review of smoking cessation interventions in the emergency setting. Am J Emerg Med. 2014;32: 713–24.. doi:10.1016/j.ajem.2014.03.042
12. Rigotti NA, Clair C, Munafò MR. Stead LF. Interventions for smoking cessation in hospitalised patients. Cochrane Database of Systematic Reviews. 2012; 5, CD001837.
13. Rice VH, Heath L, Livingstone-Banks J. Hartmann-Boyce J. Nursing interventions for smoking cessation. Cochrane Database of Systematic Reviews. 2017; 12, CD001188.
14. Lancaster, T. & Stead, L. F. Individual behavioural counselling for smoking cessation. Cochrane Database of Systematic Reviews. 3, CD001292 (2017).

15. Chan SS, Leung DY, Wong DC, Lau CP, Wong VT, Lam TH. A randomized controlled trial of stage-matched intervention for smoking cessation in cardiac out-patients. Addiction. 2012;107:829–37.
16. Katz DA, Paez MW, Reisinger HS, et al. Implementation of smoking cessation guidelines in the emergency department: a qualitative study of staff perceptions. Addict Sci Clin Pract. 2014;9(1):1.
17. Bernstein SL, Boudreaux ED, Cydulka RK, et al. Tobacco control interventions in the emergency department: a joint statement of emergency medicine organizations. J Emerg Nurs. 2006;32(5):370-381.
18. Li HCW, Wang MP, LAM TH, et al. Brief intervention to promote smoking cessation and improve glycemic control in smokers with type 2 diabetes: a randomized controlled trial. Sci Rep. 2017; 7: 45902.
19. Li HCW, Wang MP, Ho KY, et al. Helping cancer patients quit smoking using brief advice based on risk communication: A randomized controlled trial. Sci Rep. 2018, 8:2712.
20. Chan SSC, Leung DYP, Abdullah ASM, Wong VT, Hedley AJ, Lam TH. A randomized controlled trial of a smoking reduction plus nicotine replacement therapy intervention for smokers not willing to quit smoking. Addiction. 2011;106:1155–63.
21. Zhu SH, Anderson CM, Tedeschi GJ, et al. Evidence of real-world effectiveness of a telephone quitline for smokers. N Engl J Med 2002; 347: 1087e93.
22. Li WHC, Ho KY, Wang MP, et al. Effectiveness of a Brief Self-determination Theory-Based Smoking Cessation Intervention for Smokers at Emergency Departments in Hong Kong: A Randomized Clinical Trial. JAMA Intern Med. 2020;180(2):206-214
23. Ntoumanis et al. A meta-analysis of self-determination theory-informed intervention studies in the health domain: effects on motivation, health behavior, physical, and psychological health. Health Psychol Rev. 2020:3;1-31. doi: 10.1080/17437199.2020.1718529.
24. Wang MP, Suen YN, Li WH, Lam CO, Yong-da Wu S, Kwong AC, Lai VW, Chan SS, Lam TH. Intervention With Brief Cessation Advice Plus Active Referral for Proactively Recruited Community Smokers: A Pragmatic Cluster Randomized Clinical Trial. JAMA internal medicine. 2017; 177(12):1790-7.
25. Tzelepis F, Paul CL, Walsh RA, McElduff P, Knight J. Proactive telephone counseling for smoking cessation: meta-analyses by recruitment channel and methodological quality. J Natl Cancer Inst. 2011; 103 (12):922-41.
26. Tindle HA, Daigh R, Reddy VK, Bailey LA, Ochs JA, Maness MH, Davis EM, Schulze AE, Powers KM, Ylioja TE, Baca HB. eReferral between hospitals and quitlines: an emerging tobacco control strategy. Am J Prev Med. 2016; 51(4):522-6
27. Shiffman S, Ferguson SG, Strahs KR. Quitting by Gradual Smoking Reduction Using Nicotine Gum: A Randomized Controlled Trial. Am J Prev Med 2009; 36: 96-104.e101.
28. Damschroder LJ. Clarity out of chaos: Use of theory in implementation research. Psychiatry Res. 2020; 283:112461.

29. Glasgow RE, Harden SM, Gaglio B, et al. RE-AIM Planning and Evaluation Framework: Adapting to New Science and Practice With a 20-Year Review. Front Public Health. 2019;7:64.
30. Sweet SN, Ginis KA, Estabrooks PA, Latimer-Cheung AE. Operationalizing the RE-AIM framework to evaluate the impact of multi-sector partnerships. Implement Sci. 2014;9:74.
31. Glasgow RE, Vogt TM, Boles SM. Evaluating the public health impact of health promotion interventions: the RE-AIM framework. Am J Public Health. 1999;89(9):1322-1327.
32. List of all Accident & Emergency Departments. Hospital Authority. Hong Kong. https://www.ha.org.hk/visitor/ha_visitor_index.asp?Content ID=200246&lang=ENG. Accessed on 04 December 2020.
33. Piper ME, Bullen C, Krishnan-Sarin S, Rigotti NA, Steinberg ML, Streck JM, Joseph AM. Defining and Measuring Abstinence in Clinical Trials of Smoking Cessation Interventions: An Updated Review. Nicotine Tob Res. 2020 Jun 12;22(7):1098-1106. doi: 10.1093/ntr/ntz110. PMID: 31271211.
34. Dogherty EJ, Harrison MB, Graham ID. Facilitation as a role and process in achieving evidence-based practice in nursing: a focused review of concept and meaning. Worldviews Evid Based Nurs. 2010;7:76-89
35. Dhand NK, Khatkar MS. Statulator: An online statistical calculator. Sample Size Calculator for Comparing Two Paired Proportions. 2014. Accessed 7 December 2020 at http://statulator.com/SampleSize/ss2PP.html
36. Wang M. Generalized Estimating Equations in Longitudinal Data Analysis: A Review and Recent Developments. Advances in Statistics. 2014:1–11.
37. Braun V, Clarke V. Using thematic analysis in psychology. Qual Res Psychol. 2006; 3(2):77-101.
38. Michie S, Hyder N, Walia A, West R. Development of a taxonomy of behaviour change techniques used in individual behavioural support for smoking cessation. Addict Behav. 2011; 36(4):315-9.
