# Supplementary material for: Evidence-based brief cessation advice plus active referral for emergency department patients who smoke: a single-arm, real-world clinical trial
Source: BMC Med. 2025 Nov 27;23:714. doi: 10.1186/s12916-025-04534-9 (PMC12751522; doi:10.1186/s12916-025-04534-9)
Supplement: Supplementary file 3 — Additional file 3. Questionnaires-Healthcare Professionals. Baseline, post training, and 3/6-month follow-up Questionnaires. [file 12916_2025_4534_MOESM3_ESM.pdf]

Questionnaires-Healthcare Professionals
Implementation of an evidence-based smoking cessation intervention comprising brief advice plus active referrals for smokers attending emergency departments in Hong Kong

Baseline Questionnaire (Healthcare Professionals)

Part 1: Knowledge about smoking

Please read the following sentences, and then'√ ' to select the appropriate choices.

	1. Correct	2. Don't Know	3. Incorrect
A1. No matter how long a person smokes, quitting smoking is never too late	☐	☐	☐
A2. Among every two smokers, at least one would die prematurely because of smoking	☐	☐	☐
A3. Among every three smokers who started long-term heavy smoking at young age, two would die prematurely because of smoking	☐	☐	☐
A4. Nicotine patch and nicotine gum can increase the success rate of quitting smoking	☐	☐	☐
A5. Second hand smoke intensifies (outdoor) air pollution and is harmful to health	☐	☐	☐
A6. After someone smoking in a house, the tobacco residual chemicals left in the environment, for example, clothes, wall, furniture, would damage the health of infants and children	☐	☐	☐
A7. Use of e-cigarettes is harmful to human health	☐	☐	☐
A8. Use of e-cigarettes is allowed in non-smoking area	☐	☐	☐
A9. Shisha is less harmful to health than traditional rolled cigarettes	☐	☐	☐
A10. Shisha contains less nicotine than traditional rolled cigarettes	☐	☐	☐
A11. Heat-not-burn tobacco products can be addictive	☐	☐	☐
A12. Heat-not-burn tobacco products is harmful to human health	☐	☐	☐

Part 2: Attitudes towards smoking and tobacco control

Please choose to what extent you agree with the following sentences, and then'√ ' to select the appropriate choices.

	1. Strongly Agree	2. Agree	3. No Comment	4. Disagree	5. Strongly Disagree
B1. I would proactively advise friends to quit smoking	☐	☐	☐	☐	☐
B2. I would ask others not to smoke around me	☐	☐	☐	☐	☐
B3. I would remind others that they are not allowed to smoke in non-smoking area	☐	☐	☐	☐	☐
B4. I am in favor of expanding non-smoking areas	☐	☐	☐	☐	☐
B5. I am in favor of prohibiting the display of tobacco products in shops, newsstands and other places	☐	☐	☐	☐	☐
B6. I support the government to increase funding for smoking cessation services	☐	☐	☐	☐	☐
B7. I am in favor of increasing tobacco tax (If agreed, how much should the retail price of each pack of cigarettes increase to? $__) [The current retail price of major brand cigarettes is about $57 per pack]	☐	☐	☐	☐	☐
B8. I support the implementation of full warning packaging (i.e. all tobacco products must follow standardized packaging, no brand logos can be shown, brand names can onlyy be displayed on the cigarette packages in designated color, fonts and positions)	☐	☐	☐	☐	☐
B9. I support a total ban on e-cigarettes	☐	☐	☐	☐	☐
B10. I support a total ban on heat-not-burn tobacco products	☐	☐	☐	☐	☐
B11. I support a total ban on tobacco products	☐	☐	☐	☐	☐

BB1. How effective do you think is providing brief smoking cessation counselling and referrals for smokers to quit smoking?
(0 = least effective；10 = most effective): ____(0–10)

BB2. How effective do you think is providing brief smoking cessation counselling and referrals in helping smokers to quit smoking?
(0 = least effective；10 = most effective): ____(0–10)

Part 3: Past experience in providing brief cessation counselling and referral to smokers to quit smoking

C1. Among the people who live with you, who is a smoker? (Can select multiple options)

a ☐ None   b ☐ Father   c ☐ Mother   d ☐ Brother (s)   e ☐ Sister (s)

f ☐ Grandfather   g ☐ Grandmother   h ☐ Other relatives   i ☐ Domestic helper   j ☐ Others: ____________

C2. Have you inhaled second-hand smoke at home within past one week? 0.☐ No 1.☐ Yes, ___days (1-7)

C3. Have you inhaled second-hand smoke in other places (e.g. public places, bus station) within past one week?

0.☐ No 1.☐ Yes, ___days (1-7)

C4. Have you asked others not to smoke around you **within past six months**? 0.☐ No 1.☐ Yes, ___times 2.☐N/A

C5. Have you reminded others not to smoke in non-smoking areas **within past six months**?

0.☐ No 1.☐ Yes, ___times 2.☐N/A

C6. Have you ever offered brief smoking cessation counselling to any smokers (including people living with you or friends) **within past six months**?

1.☐ Yes   0.☐ No ***(jump to C7)***

C6a. To how many smokers have you given brief smoking cessation counselling **within past six months**? _____

C7i. How many smokers who attended the AED (in the hospital where you work) have you encountered **within past six months**? _____

C7. Have you ever provided brief smoking cessation counselling to smokers who attended the AED (in the hospital where you work) **within past six months**?

1.☐ Yes   0.☐ No ***(jump to C7b)***

C7a. To how many smokers who attended the AED (in the hospital where you work) have you given brief smoking cessation counselling **within past six months**? _____

C7b. Have you referred smokers who attended the AED (in the hospital where you work) to participate in smoking cessation counselling/services provided by the government, university or other institutions **within past six months**?

1.☐ Yes, ____ (Number of Smokers), ____________________ (Institution Name)

____ (Number of Smokers), ____________________ (Institution Name)

____ (Number of Smokers), ____________________ (Institution Name)

____ (Number of Smokers), ____________________ (Institution Name)

____ (Number of Smokers), ____________________ (Institution Name) ***(jump to C7d)*** 0.☐ No ***(jump to C7c)***

C7c. Why did you not refer smokers to participate in smoking cessation counselling/services provided by the government, universities or other institutions? Please'√ ' to select the appropriate choice(s). (Can select multiple options)

a ☐ Did not know that these institutions provide smoking cessation counselling/services

b ☐ Not clear on how to contact these institutions

c ☐ Not clear on the content of the smoking cessation counselling/services offered by these institutions

d ☐ Not clear on who these institutions serve

e ☐ Not clear on whether the smoking cessation counselling/services offered by these institutions are free or not

f ☐ Not clear on the effectiveness of the smoking cessation counselling/services offered by these institutions

g ☐ Complicated referral process

h ☐ Lack of time

i ☐ No reason

j ☐ Have not encountered any smokers

k ☐ Others (Please specify:__________________)

C7d. When you try to refer smokers to quit smoking/provide brief smoking cessation counselling, which of the following would **hinder** you? Please'√ ' to select the appropriate choice(s). (Can select multiple options)

a ☐ Lack of time

b ☐ Lack of communication and counselling techniques

c ☐ Lack of knowledge about smoking cessation counselling/services/promotion

d ☐ Lack of knowledge about tobacco and health

e ☐ Lack of confidence in providing smoke cessation counselling/services/promotion

f ☐ Lack of support from friends/families/supervisors/others

g ☐ Lack of support from experienced smoking cessation professionals

h ☐ Fear of smokers' refusal to accept smoking cessation counselling

i ☐ Belief in smoking is a way for smokers to cope with stress

j ☐ Inability in helping smokers to deal with daily-life problems

k ☐ Lack of contact with smokers

l ☐ No responsibility in providing smoking cessation advice/promoting smoke-free messages

m ☐ Belief in the ineffectiveness of providing smoking cessation advice/promoting smoke-free messages
n ☐ No encounter with smokers
o ☐ Without any hindrance
p ☐ Others (Please specify: ____________________)

Part 4: Self-efficacy in providing brief cessation counselling and referring smokers to quit smoking

D1. You regard yourself having how much confidence in providing brief smoking cessation counselling and referring smokers to quit smoking? (0 = No confidence; 10 = Full confidence): ____ (0-10)

D2. How important do you think brief smoking cessation counselling and referrals are in helping smokers quit smoking? (0 = Totally insignificant; 10 = Extremely important): ____ (0-10)

D3. How difficult do you think it would be when you provide brief smoking cessation counselling and referrals? (0 = Not difficult at all; 10 = extremely difficult): ____ (0-10)

Part 5: Provide brief cessation counseling and referral to smokers in the future

E1. Do you intend to provide brief smoking cessation counselling and referrals to smokers in the future?
1.☐Yes ***(jump to E2)*** 0.☐No

E1a. Why not intended to do so? Please'√ ' to select the appropriate choice(s). (Can select multiple options)

a ☐ Lack of time
b ☐ Lack of communication and counselling techniques
c ☐ Lack of knowledge about smoking cessation counselling/services/promotion
d ☐ Lack of knowledge about tobacco and health
e ☐ Lack of confidence in providing smoke cessation counselling/services/promotion
f ☐ Lack of support from friends/families/supervisors/others
g ☐ Lack of support from experienced smoking cessation professionals
h ☐ Fear of smokers' refusal to accept smoking cessation counselling
i ☐ Belief in smoking is a way for smokers to cope with stress
j ☐ Inability in helping smokers to deal with daily-life problems
k ☐ Lack of contact with smokers
l m ☐ No responsibility in providing smoking cessation advice/promoting smoke-free messages
n ☐ Belief in the ineffectiveness of providing smoking cessation advice/promoting smoke-free messages
c p ☐ No encounter with smokers
q ☐ Without any hindrance
r s ☐ Others (Please specify: ____________________)

E2. Would you provide brief smoking cessation counselling and referrals to smokers who attend AED (in the hospital where you work) in the future? 1. ☐Yes ***(jump to E3)*** 0. ☐No

E2a. Why not? Please '√ ' to select the appropriate choice(s). (Can select multiple options)

a ☐ Lack of time
b ☐ Lack of communication and counselling techniques
c ☐ Lack of knowledge about smoking cessation counselling/services/promotion
d ☐ Lack of knowledge about tobacco and health
e ☐ Lack of confidence in providing smoke cessation counselling/services/promotion
f ☐ Lack of support from friends/families/supervisors/others
g ☐ Lack of support from experienced smoking cessation professionals
h ☐ Fear of smokers' refusal to accept smoking cessation counselling
i ☐ Belief in smoking is a way for smokers to cope with stress
j ☐ Inability in helping smokers to deal with daily-life problems
k ☐ Lack of contact with smokers
l ☐ No responsibility in providing smoking cessation advice/promoting smoke-free messages
m ☐ Belief in the ineffectiveness of providing smoking cessation advice/promoting smoke-free messages
n ☐ No encounter with smokers
o ☐ Without any hindrance
p ☐ Others (Please specify: ____________________)

E3. Would you provide brief smoking cessation counselling and referrals to any smokers in the future?
1. ☐ Yes ***(jump to Part 6: Personal Information)*** 0. ☐ No

E3a. Why not? Please '√ ' to select the appropriate choice(s). (Can select multiple options)

a ☐ Lack of time
b ☐ Lack of communication and counselling techniques
c ☐ Lack of knowledge about smoking cessation counselling/services/promotion
d ☐ Lack of knowledge about tobacco and health
e ☐ Lack of confidence in providing smoke cessation counselling/services/promotion
f ☐ Lack of support from friends/families/supervisors/others

g ☐ Lack of support from experienced smoking cessation professionals

h ☐ Fear of smokers' refusal to accept smoking cessation counselling

i ☐ Belief in smoking is a way for smokers to cope with stress

j ☐ Inability in helping smokers to deal with daily-life problems

k ☐ Lack of contact with smokers

l m ☐ No responsibility in providing smoking cessation advice/promoting smoke-free messages

n ☐ Belief in the ineffectiveness of providing smoking cessation advice/promoting smoke-free messages

c p ☐ No encounter with smokers

q ☐ Without any hindrance

r s ☐ Others (Please specify: ____________________)

Part 6: Personal Information

Please provide the following information for the analysis and research of this survey:

* You 1. ☐ Never smoked 2. ☐ Are currently smoking 3. ☐ Used to smoke, but now quit

Name：_________________________ Gender：M / F Age：_______________

Hospital：_______________________ Position：__________________________

~ End of Questionnaire ~

Serial number :____________

Implementation of an evidence-based smoking cessation intervention comprising brief advice plus active referrals for smokers attending emergency departments in Hong Kong

Post training (Healthcare Professionals)

Part 1: Knowledge about smoking

Please read the following sentences, and then'√ ' to select the appropriate choices.

	1. Correct	2. Don't Know	3. Incorrect
A1. No matter how long a person smokes, quitting smoking is never too late	☐	☐	☐
A2. Among every two smokers, at least one would die prematurely because of smoking	☐	☐	☐
A3. Among every three smokers who started long-term heavy smoking at young age, two would die prematurely because of smoking	☐	☐	☐
A4. Nicotine patch and nicotine gum can increase the success rate of quitting smoking	☐	☐	☐
A5. Second hand smoke intensifies (outdoor) air pollution and is harmful to health	☐	☐	☐
A6. After someone smoking in a house, the tobacco residual chemicals left in the environment, for example, clothes, wall, furniture, would damage the health of infants and children	☐	☐	☐
A7. Use of e-cigarettes is harmful to human health	☐	☐	☐
A8. Use of e-cigarettes is allowed in non-smoking area	☐	☐	☐
A9. Shisha is less harmful to health than traditional rolled cigarettes	☐	☐	☐
A10. Shisha contains less nicotine than traditional rolled cigarettes	☐	☐	☐
A11. Heat-not-burn tobacco products can be addictive	☐	☐	☐
A12. Heat-not-burn tobacco products is harmful to human health	☐	☐	☐

Part 2: Attitudes towards smoking and tobacco control

Please choose to what extent you agree with the following sentences, and then'√ ' to select the appropriate choices.

	1. Strongly Agree	2. Agree	3. No Comment	4. Disagree	5. Strongly Disagree
B1. I would proactively advise friends to quit smoking	☐	☐	☐	☐	☐
B2. I would ask others not to smoke around me	☐	☐	☐	☐	☐
B3. I would remind others that they are not allowed to smoke in non-smoking area	☐	☐	☐	☐	☐
B4. I am in favor of expanding non-smoking areas	☐	☐	☐	☐	☐
B5. I am in favor of prohibiting the display of tobacco products in shops, newsstands and other places	☐	☐	☐	☐	☐
B6. I support the government to increase funding for smoking cessation services	☐	☐	☐	☐	☐
B7. I am in favor of increasing tobacco tax (If agreed, how much should the retail price of each pack of cigarettes increase to? $__) [The current retail price of major brand cigarettes is about $57 per pack]	☐	☐	☐	☐	☐
B8. I support the implementation of full warning packaging (i.e. all tobacco products must follow standardized packaging, no brand logos can be shown, brand names can onlyy be displayed on the cigarette packages in designated color, fonts and positions)	☐	☐	☐	☐	☐
B9. I support a total ban on e-cigarettes	☐	☐	☐	☐	☐
B10. I support a total ban on heat-not-burn tobacco products	☐	☐	☐	☐	☐
B11. I support a total ban on tobacco products	☐	☐	☐	☐	☐

BB1. How effective do you think is providing brief smoking cessation counselling and referrals for smokers to quit smoking?
(0 = least effective；10 = most effective): ____(0–10)

BB2. How effective do you think is providing brief smoking cessation counselling and referrals in helping smokers to quit smoking?
(0 = least effective；10 = most effective): ____(0–10)

Part 3: Self-efficacy in providing brief cessation counselling and referring smokers to quit smoking

D1. You regard yourself having how much confidence in providing brief smoking cessation counselling and referring smokers to quit smoking? (0 = No confidence; 10 = Full confidence): ____ (0-10)

D2. How important do you think brief smoking cessation counselling and referrals are in helping smokers quit smoking?
(0 = Totally insignificant; 10 = Extremely important): ____ (0-10)

D3. How difficult do you think it would be when you provide brief smoking cessation counselling and referrals?
(0 = Not difficult at all; 10 = extremely difficult): ____ (0-10)

Part 4: Provide brief cessation counseling and referral to smokers in the future

E1. Do you intend to provide brief smoking cessation counselling and referrals to smokers in the future?
1.☐ Yes ***(jump to E2)*** 0.☐ No

E1a. Why not intended to do so? Please'√ ' to select the appropriate choice(s). (Can select multiple options)

a	☐ Lack of time	b	☐ Lack of communication and counselling techniques
c	☐ Lack of knowledge about smoking cessation counselling/services/promotion	d	☐ Lack of knowledge about tobacco and health
e	☐ Lack of confidence in providing smoke cessation counselling/services/promotion	f	☐ Lack of support from friends/families/supervisors/others
g	☐ Lack of support from experienced smoking cessation professionals	h	☐ Fear of smokers' refusal to accept smoking cessation counselling
i	☐ Belief in smoking is a way for smokers to cope with stress	j	☐ Inability in helping smokers to deal with daily-life problems
k	☐ Lack of contact with smokers	l m	☐ No responsibility in providing smoking cessation advice/promoting smoke-free messages
n	☐ Belief in the ineffectiveness of providing smoking cessation advice/promoting smoke-free messages	c p	☐ No encounter with smokers
q	☐ Without any hindrance	r s	☐ Others (Please specify: ____________________)

E2. Would you provide brief smoking cessation counselling and referrals to smokers who attend AED (in the hospital where you work) in the future? 1. ☐ Yes ***(jump to E3)*** 0. ☐ No

E2a. Why not? Please '√ ' to select the appropriate choice(s). (Can select multiple options)

a	☐ Lack of time	b	☐ Lack of communication and counselling techniques
c	☐ Lack of knowledge about smoking cessation counselling/services/promotion	d	☐ Lack of knowledge about tobacco and health
e	☐ Lack of confidence in providing smoke cessation counselling/services/promotion	f	☐ Lack of support from friends/families/supervisors/others
g	☐ Lack of support from experienced smoking cessation professionals	h	☐ Fear of smokers' refusal to accept smoking cessation counselling
i	☐ Belief in smoking is a way for smokers to cope with stress	j	☐ Inability in helping smokers to deal with daily-life problems
k	☐ Lack of contact with smokers	l m	☐ No responsibility in providing smoking cessation advice/promoting smoke-free messages
n	☐ Belief in the ineffectiveness of providing smoking cessation advice/promoting smoke-free messages	c p	☐ No encounter with smokers
q	☐ Without any hindrance	r s	☐ Others (Please specify: ____________________)

E3. Would you provide brief smoking cessation counselling and referrals to any smokers in the future?
1. ☐ Yes ***(jump to Part 5: Opinions on this brief smoking cessation counseling workshop)*** 0. ☐ No

E3a. Why not? Please '√ ' to select the appropriate choice(s). (Can select multiple options)

a	☐ Lack of time	b	☐ Lack of communication and counselling techniques
c	☐ Lack of knowledge about smoking cessation counselling/services/promotion	d	☐ Lack of knowledge about tobacco and health
e	☐ Lack of confidence in providing smoke cessation counselling/services/promotion	f	☐ Lack of support from friends/families/supervisors/others
g	☐ Lack of support from experienced smoking cessation professionals	h	☐ Fear of smokers' refusal to accept smoking cessation counselling
i	☐ Belief in smoking is a way for smokers to cope with stress	j	☐ Inability in helping smokers to deal with daily-life problems
k	☐ Lack of contact with smokers	l	☐ No responsibility in providing smoking cessation advice/promoting smoke-free messages
m	☐ Belief in the ineffectiveness of providing smoking cessation advice/promoting smoke-free messages	n	☐ No encounter with smokers
o	☐ Without any hindrance	p	Others (Please specify: ____________________)

Part5: Opinions on this brief smoking cessation counseling workshop

Please choose to what extent you agree with the following sentences, and then'√ ' to select the appropriate choices.

	1. Strongly Agree	2. Agree	3. No Comment	4. Disagree	5. Strongly Disagree
F1. The content of the workshop was enriching	☐	☐	☐	☐	☐
F2. The environment and facilities of the workshop was comprehensive	☐	☐	☐	☐	☐
F3. The time arrangement of the workshop was ideal	☐	☐	☐	☐	☐
F4. The workshop helped me to develop independent and critical thinking ability	☐	☐	☐	☐	☐
F5. The workshop helped me to improve communication skills	☐	☐	☐	☐	☐
F6. The workshop helped me to improve problem-solving abilities	☐	☐	☐	☐	☐
F7. The workshop helped me grasp smoking cessation councelling techniques	☐	☐	☐	☐	☐
F8. The workshop made me understand the advantages of a smoke-free lifestyle	☐	☐	☐	☐	☐
F9. The workshop has eliminated the obstacles I encountered when offering brief smoking cessation counselling and referrals	☐	☐	☐	☐	☐
F10. The workshop helped me understand the harm of smoking	☐	☐	☐	☐	☐
F11. The workshop helped me grasp the information related to the smoking cessation counselling/services offered by other institutions, including content, fees, target groups, etc.	☐	☐	☐	☐	☐
F12. The workshop raised my confidence in offering brief smoking cessation counselling and referrals	☐	☐	☐	☐	☐
F13. The workshop helped me to offer brief smoking cessation counselling and referrals in my working environment	☐	☐	☐	☐	☐
F14. The workshop helped me to offer brief smoking cessation counselling and referrals in the community	☐	☐	☐	☐	☐
F15. The information taught by the workshop tutor was very suitable for me	☐	☐	☐	☐	☐
F16. The workshop tutor knows very well about smoking cessation counselling	☐	☐	☐	☐	☐
F17. The content and activities of the workshop have fully met my expectations and targets	☐	☐	☐	☐	☐
F18. I would introduce this workshop to friends/colleagues	☐	☐	☐	☐	☐
F19. Overall, I am satisfied with this workshop	☐	☐	☐	☐	☐

F20. If there is a new workshop next time, do you want to participate?

1. ☐ Really want to participate 2. ☐ want to participate 3. ☐ Undecided 4. ☐ don't want to participate 5. ☐ Strongly want to participate

F21. What activities do you think should be increased/decreased in the workshop?

____

F22. If you have any other valuable comments on the workshop, please provide:

____

Part 6: Personal Information

Please provide the following information for the analysis and research of this survey:

＊ You 1. ☐ Never smoked 2. ☐ Are currently smoking 3. ☐ Used to smoke, but now quit

Name: __________________________ Gender: M / F Telephone no.: _______________

Hospital: ________________________ Position: _________________________ Age: _________

~ End of Questionnaire ~

Serial number :____________

Implementation of an evidence-based smoking cessation intervention comprising brief advice plus active referrals for smokers attending emergency departments in Hong Kong

3/6-month follow-up Questionnaire (Healthcare Professionals)

Part 1: Knowledge about smoking

Please read the following sentences, and then'√ ' to select the appropriate choices.

	1. Correct	2. Don't Know	3. Incorrect
A1. No matter how long a person smokes, quitting smoking is never too late	☐	☐	☐
A2. Among every two smokers, at least one would die prematurely because of smoking	☐	☐	☐
A3. Among every three smokers who started long-term heavy smoking at young age, two would die prematurely because of smoking	☐	☐	☐
A4. Nicotine patch and nicotine gum can increase the success rate of quitting smoking	☐	☐	☐
A5. Second hand smoke intensifies (outdoor) air pollution and is harmful to health	☐	☐	☐
A6. After someone smoking in a house, the tobacco residual chemicals left in the environment, for example, clothes, wall, furniture, would damage the health of infants and children	☐	☐	☐
A7. Use of e-cigarettes is harmful to human health	☐	☐	☐
A8. Use of e-cigarettes is allowed in non-smoking area	☐	☐	☐
A9. Shisha is less harmful to health than traditional rolled cigarettes	☐	☐	☐
A10. Shisha contains less nicotine than traditional rolled cigarettes	☐	☐	☐
A11. Heat-not-burn tobacco products can be addictive	☐	☐	☐
A12. Heat-not-burn tobacco products is harmful to human health	☐	☐	☐

Part 2: Attitudes towards smoking and tobacco control

Please choose to what extent you agree with the following sentences, and then'√ ' to select the appropriate choices.

	1. Strongly Agree	2. Agree	3. No Comment	4. Disagree	5. Strongly Disagree
B1. I would proactively advise friends to quit smoking	☐	☐	☐	☐	☐
B2. I would ask others not to smoke around me	☐	☐	☐	☐	☐
B3. I would remind others that they are not allowed to smoke in non-smoking area	☐	☐	☐	☐	☐
B4. I am in favor of expanding non-smoking areas	☐	☐	☐	☐	☐
B5. I am in favor of prohibiting the display of tobacco products in shops, newsstands and other places	☐	☐	☐	☐	☐
B6. I support the government to increase funding for smoking cessation services	☐	☐	☐	☐	☐
B7. I am in favor of increasing tobacco tax (If agreed, how much should the retail price of each pack of cigarettes increase to? $__) [The current retail price of major brand cigarettes is about $57 per pack]	☐	☐	☐	☐	☐
B8. I support the implementation of full warning packaging (i.e. all tobacco products must follow standardized packaging, no brand logos can be shown, brand names can onlyy be displayed on the cigarette packages in designated color, fonts and positions)	☐	☐	☐	☐	☐
B9. I support a total ban on e-cigarettes	☐	☐	☐	☐	☐
B10. I support a total ban on heat-not-burn tobacco products	☐	☐	☐	☐	☐
B11. I support a total ban on tobacco products	☐	☐	☐	☐	☐

BB1. How effective do you think is providing brief smoking cessation counselling and referrals for smokers to quit smoking?
(0 = least effective；10 = most effective): ____(0–10)

BB2. How effective do you think is providing brief smoking cessation counselling and referrals in helping smokers to quit smoking?
(0 = least effective；10 = most effective): ____(0–10)

第三部份: <u>過去</u>提供簡短戒煙輔導及轉介吸煙者戒煙的經驗

C1. Among the people who live with you, who is a smoker? (Can select multiple options)

a ☐ None b ☐ Father c ☐ Mother d ☐ Brother (s) e ☐ Sister (s)

f ☐ Grandfather g ☐ Grandmother h ☐ Other relatives i ☐ Domestic helper j ☐ Others: ____________

C2. Have you inhaled second-hand smoke at home within past one week? 0.☐ No 1.☐ Yes, ___days (1-7)

C3. Have you inhaled second-hand smoke in other places (e.g. public places, bus station) within past one week?

0.☐ No 1.☐ Yes, ___days (1-7)

C4. Have you asked others not to smoke around you **within past three months**? 0.☐ No 1.☐ Yes, ___times 2.☐N/A

C5. Have you reminded others not to smoke in non-smoking areas **within past three months**?

0.☐ No 1.☐ Yes, ___times 2.☐N/A

C6. Have you ever offered brief smoking cessation counselling to any smokers (including people living with you or friends) **within past three months**?

1.☐ Yes 0.☐ No ***(jump to C7)***

C6a. To how many smokers have you given brief smoking cessation counselling **within past three months**? _____
How many smokers who attended the AED (in the hospital where you work) have you encountered **within past three months**? _____

C7. Have you ever provided brief smoking cessation counselling to smokers who attended the AED (in the hospital where you work) **within past three months**?

1.☐ Yes 0.☐ No ***(jump to C7b)***

C7a. To how many smokers who attended the AED (in the hospital where you work) have you given brief smoking cessation counselling **within past three months**? _____

C7b. Have you referred smokers who attended the AED (in the hospital where you work) to participate in smoking cessation counselling/services provided by the government, university or other institutions **within past three months**?

1.☐ Yes, ____ (Number of Smokers), ____________________ (Institution Name)

____ (Number of Smokers), ____________________ (Institution Name)

____ (Number of Smokers), ____________________ (Institution Name)

____ (Number of Smokers), ____________________ (Institution Name)

____ (Number of Smokers), ____________________ (Institution Name) ***(jump to C7d)*** 0.☐ No ***(jump to C7c)***

C7c. Why did you not refer smokers to participate in smoking cessation counselling/services provided by the government, universities or other institutions? Please'√ ' to select the appropriate choice(s). (Can select multiple options)

a ☐ Did not know that these institutions provide smoking cessation counselling/services
b ☐ Not clear on how to contact these institutions
c ☐ Not clear on the content of the smoking cessation counselling/services offered by these institutions
d ☐ Not clear on who these institutions serve
e ☐ Not clear on whether the smoking cessation counselling/services offered by these institutions are free or not
f ☐ Not clear on the effectiveness of the smoking cessation counselling/services offered by these institutions
g ☐ Complicated referral process
h ☐ Lack of time
i ☐ No reason
j ☐ Have not encountered any smokers
k ☐ Others (Please specify:__________________)

C7d. When you try to refer smokers to quit smoking/provide brief smoking cessation counselling, which of the following would **hinder** you? Please'√ ' to select the appropriate choice(s). (Can select multiple options)

a ☐ Lack of time
b ☐ Lack of communication and counselling techniques
c ☐ Lack of knowledge about smoking cessation counselling/services/promotion
d ☐ Lack of knowledge about tobacco and health
e ☐ Lack of confidence in providing smoke cessation counselling/services/promotion
f ☐ Lack of support from friends/families/supervisors/others
g ☐ Lack of support from experienced smoking cessation professionals
h ☐ Fear of smokers' refusal to accept smoking cessation counselling
i ☐ Belief in smoking is a way for smokers to cope with stress
j ☐ Inability in helping smokers to deal with daily-life problems
k ☐ Lack of contact with smokers
l ☐ No responsibility in providing smoking cessation advice/promoting smoke-free messages
m ☐ Belief in the ineffectiveness of providing smoking
n ☐ No encounter with smokers

cessation advice/promoting smoke-free messages

o ☐ Without any hindrance

p ☐ Others (Please specify: ____________________)

Part 4: Self-efficacy in providing brief cessation counselling and referring smokers to quit smoking

D1. You regard yourself having how much confidence in providing brief smoking cessation counselling and referring smokers to quit smoking? (0 = No confidence; 10 = Full confidence): ____ (0-10)

D2. How important do you think brief smoking cessation counselling and referrals are in helping smokers quit smoking? (0 = Totally insignificant; 10 = Extremely important): ____ (0-10)

D3. How difficult do you think it would be when you provide brief smoking cessation counselling and referrals? (0 = Not difficult at all; 10 = extremely difficult): ____ (0-10)

Part 5: Provide brief cessation counseling and referral to smokers in the future

E1. Do you intend to provide brief smoking cessation counselling and referrals to smokers in the future?

1.☐Yes ***(jump to E2)*** 0.☐No

E1a. Why not intended to do so? Please'√ ' to select the appropriate choice(s). (<u>Can select multiple options</u>)

a ☐ Lack of time

b ☐ Lack of communication and counselling techniques

c ☐ Lack of knowledge about smoking cessation counselling/services/promotion

d ☐ Lack of knowledge about tobacco and health

e ☐ Lack of confidence in providing smoke cessation counselling/services/promotion

f ☐ Lack of support from friends/families/supervisors/others

g ☐ Lack of support from experienced smoking cessation professionals

h ☐ Fear of smokers' refusal to accept smoking cessation counselling

i ☐ Belief in smoking is a way for smokers to cope with stress

j ☐ Inability in helping smokers to deal with daily-life problems

k ☐ Lack of contact with smokers

l ☐ No responsibility in providing smoking cessation advice/promoting smoke-free messages

m ☐ Belief in the ineffectiveness of providing smoking cessation advice/promoting smoke-free messages

n ☐ No encounter with smokers

o ☐ Without any hindrance

p ☐ Others (Please specify: ____________________)

E2. Would you provide brief smoking cessation counselling and referrals to smokers who attend AED (in the hospital where you work) in the future? 1. ☐Yes ***(jump to E3)*** 0. ☐No

E2a. Why not? Please '√ ' to select the appropriate choice(s). (<u>Can select multiple options</u>)

a ☐ Lack of time

b ☐ Lack of communication and counselling techniques

c ☐ Lack of knowledge about smoking cessation counselling/services/promotion

d ☐ Lack of knowledge about tobacco and health

e ☐ Lack of confidence in providing smoke cessation counselling/services/promotion

f ☐ Lack of support from friends/families/supervisors/others

g ☐ Lack of support from experienced smoking cessation professionals

h ☐ Fear of smokers' refusal to accept smoking cessation counselling

i ☐ Belief in smoking is a way for smokers to cope with stress

j ☐ Inability in helping smokers to deal with daily-life problems

k ☐ Lack of contact with smokers

l m ☐ No responsibility in providing smoking cessation advice/promoting smoke-free messages

n ☐ Belief in the ineffectiveness of providing smoking cessation advice/promoting smoke-free messages

c p ☐ No encounter with smokers

q ☐ Without any hindrance

r s ☐ Others (Please specify: ____________________)

E3. Would you provide brief smoking cessation counselling and referrals to any smokers in the future?

1. ☐ Yes ***(jump to Part 6: Opinions on this brief smoking cessation counseling workshop)*** 0. ☐ No

E3a. Why not? Please '√ ' to select the appropriate choice(s). (<u>Can select multiple options</u>)

a ☐ Lack of time

b ☐ Lack of communication and counselling techniques

c ☐ Lack of knowledge about smoking cessation counselling/services/promotion

d ☐ Lack of knowledge about tobacco and health

e ☐ Lack of confidence in providing smoke cessation counselling/services/promotion

f ☐ Lack of support from friends/families/supervisors/others

g ☐ Lack of support from experienced smoking cessation professionals

h ☐ Fear of smokers' refusal to accept smoking cessation counselling

i ☐ Belief in smoking is a way for smokers to cope with stress

j ☐ Inability in helping smokers to deal with daily-life problems

k ☐ Lack of contact with smokers

l ☐ No responsibility in providing smoking cessation

m ☐ Belief in the ineffectiveness of providing smoking cessation advice/promoting smoke-free messages

advice/promoting smoke-free messages

n ☐ No encounter with smokers

o ☐ Without any hindrance

p Others (Please specify: ____________________)

Part6: Opinions on this brief smoking cessation counseling workshop

Please choose to what extent you agree with the following sentences, and then'√ ' to select the appropriate choices.

	1. Strongly Agree	2. Agree	3. No Comment	4. Disagree	5. Strongly Disagree
F4. The workshop helped me to develop independent and critical thinking ability	☐	☐	☐	☐	☐
F5. The workshop helped me to improve communication skills	☐	☐	☐	☐	☐
F6. The workshop helped me to improve problem-solving abilities	☐	☐	☐	☐	☐
F7. The workshop helped me grasp smoking cessation councelling techniques	☐	☐	☐	☐	☐
F8. The workshop made me understand the advantages of a smoke-free lifestyle	☐	☐	☐	☐	☐
F9. The workshop has eliminated the obstacles I encountered when offering brief smoking cessation counselling and referrals	☐	☐	☐	☐	☐
F10. The workshop helped me understand the harm of smoking	☐	☐	☐	☐	☐
F11. The workshop helped me grasp the information related to the smoking cessation counselling/services offered by other institutions, including content, fees, target groups, etc.	☐	☐	☐	☐	☐
F12. The workshop raised my confidence in offering brief smoking cessation counselling and referrals	☐	☐	☐	☐	☐
F13. The workshop helped me to offer brief smoking cessation counselling and referrals in my working environment	☐	☐	☐	☐	☐
F14. The workshop helped me to offer brief smoking cessation counselling and referrals in the community	☐	☐	☐	☐	☐

Part 7: Personal Information

Please provide the following information for the analysis and research of this survey:

∗ You 1. ☐ Never smoked 2. ☐ Are currently smoking 3. ☐ Used to smoke, but now quit

Name: ________________________ Gender: M / F Telephone no.: ______________

Hospital: ______________________ Position: _______________________ Age: ________

~ End of Questionnaire ~

Serial number :____________
