# Supplementary material for: Evidence-based brief cessation advice plus active referral for emergency department patients who smoke: a single-arm, real-world clinical trial
Source: BMC Med. 2025 Nov 27;23:714. doi: 10.1186/s12916-025-04534-9 (PMC12751522; doi:10.1186/s12916-025-04534-9)
Supplement: Supplementary file 4 — Additional file 4. Questionnaires-Patients. Baseline and 6/12-month follow-up Questionnaires. [file 12916_2025_4534_MOESM4_ESM.pdf]

Questionnaires-Patients
Implementation of an evidence-based smoking cessation intervention comprising brief advice plus active referrals for smokers attending emergency departments in Hong Kong

Baseline Questionnaire (Patients)

Date: ____________________________ Serial no: ________________________

A. Smoking record

A1. For how many days have you smoked traditional cigarettes within the past 7 days? _______day(s)

A2. For how many days have you smoked traditional cigarettes within the past 30 days? _______day(s)

A3. How many cigarettes did you smoke per day during Monday to Friday within the past 30 days? _______day(s)

A4. How many cigarettes did you smoke per day during weekends or holidays within the past 30 days? _______day(s)

A5. What is your age when you start smoking every week? _______

A6. Have you ever tried other tobacco products (e.g. e-cigarettes, shisha, cigars, rolled cigarettes, tobacco pipes, heat-not-burn tobacco products), even one drag counts?

1. ☐ yes   2. ☐ no ***(jump to A8)***

A6a. Have you tried other tobacco products (e.g. e-cigarettes, shisha, cigars, rolled cigarettes, tobacco pipes, heat-not-burn tobacco products) within past 7 days, even one drag counts?

1. ☐ yes   2. ☐ no

A6b. Have you tried other tobacco products (e.g. e-cigarettes, shisha, cigars, rolled cigarettes, tobacco pipes, heat-not-burn tobacco products) within past 30 days, even one drag counts?

1. ☐ yes   2. ☐ no

A7. How often did you use the following tobacco products?

	Never	Have tried	1 day per year or less	1 day per month or less	1 day per month	2-3 days per month	1-3 days per week	4-6 days per week	Everyday	**Minutes, Rolls, E-liquids, Cartridges…**
1. E-cigarettes: **(___day(s)/past 30 days)**	0 ☐	8 ☐	1 ☐	2 ☐	3 ☐	4 ☐	5 ☐	6 ☐	7 ☐	
2. Shisha: **(___day(s)/past 30 days)**	0 ☐	8 ☐	1 ☐	2 ☐	3 ☐	4 ☐	5 ☐	6 ☐	7 ☐	
3. Cigars: **(___day(s)/past 30 days)**	0 ☐	8 ☐	1 ☐	2 ☐	3 ☐	4 ☐	5 ☐	6 ☐	7 ☐	
4. Rolled cigarettes: **(___day(s)/past 30 days)**	0 ☐	8 ☐	1 ☐	2 ☐	3 ☐	4 ☐	5 ☐	6 ☐	7 ☐	
5. Tobacco pipes: **(___day(s)/past 30 days)**	0 ☐	8 ☐	1 ☐	2 ☐	3 ☐	4 ☐	5 ☐	6 ☐	7 ☐	
5a. Heat-not-burn tobacco products (e.g. IQOS): **(___day(s)/past 30 days)**	0 ☐	8 ☐	1 ☐	2 ☐	3 ☐	4 ☐	5 ☐	6 ☐	7 ☐	
6. Others: **(___day(s)/past 30 days)**	0 ☐	8 ☐	1 ☐	2 ☐	3 ☐	4 ☐	5 ☐	6 ☐	7 ☐	

Within past 30 days:

A8. How soon after you wake up did you smoke your first cigarette?

1.☐ Within 5 minutes (3 points) 2.☐ 6 to 30 minutes (2 points)

3.☐ 31 to 60 minutes (1 point) 4.☐ After 60 minutes (0 points)

A9. Did you find it difficult to refrain from smoking in places where it is forbidden (e.g. shopping malls, MTR, lifts)?

1.☐ Yes (1 point) 2.☐ No (0 points)

A10. Which cigarette would you find the most difficult to give up?

1.☐ The first cigarette in the morning (1 point) 2.☐ Others (0 points)

A11. Did you smoke more frequently during the first few hours after awakening than during the rest of the day?

1.☐ Yes (1 point) 2.☐ No (0 points)

A12. Would you smoke even when you are so ill that you are in bed most of the day?

1.☐ Yes (1 point) 2.☐ No (0 points)

A13. Cigarettes per day: ______

1.☐ 31 or above (3 points) 2.☐ 21 to 30 (2 points) 3.☐ 11 to 20 (1 point) 4.☐ 10 or less (0 points)

A13a. To be completed by staff: Total score: _______

A13b. Nicotine Dependency: 1.☐ Mild (0–3 points) 2.☐ Moderate (4–5 points) 3.☐ Severe (6-10 points)

B. Smoking cessation/reduction records

B1. Have you ever tried to **quit smoking seriously** (i.e. actively stop smoking for 24 hours or more) since you smoked? (Due to illness or camping/travelling is not counted)

1.☐ Yes 2.☐ No

B2. Have you tried to **quit smoking seriously** (i.e. actively stop smoking for 24 hours or more) in the past 1 year? (Due to illness or camping/travelling is not counted)

1.☐ Yes 2.☐ No

C. Current decision related to quitting smoking

C1. Do you plan to quit smoking now? 1.☐ Yes 2.☐ No (Pre-contemplation) ***(jump to D1)***

C2. When do you plan to start quitting smoking? **(Choose one answer only)**

0. ☐ I have stopped smoking currently (action)
1. ☐ I would quit smoking within 7 days (preparation/contemplation)
2. ☐ I would quit smoking within 1 month (preparation/contemplation)
3. ☐ I would quit smoking within 6 months (contemplation)
4. ☐ I would quit smoking after 6months (pre-contemplation)
5. ☐ Not yet decided (pre-contemplation)

C2a. 1. ☐ Decided date to quit smoking: _________________ 2. ☐ Not yet decided the exact date to quit smoking

C3. Stage of readiness to quit smoking to be completed by staff:

4. ☐ Action 3. ☐ Preparation# 2. ☐ Contemplation# 1. ☐ Pre-contemplation

Preparation: At least one 24-hour quit attempt in the past year; **Contemplation:** No quit attempt

D. Self-efficacy

D1. How important is successful quitting smoking to you? (0 = not important at all, 10 = extremely important) ____

D2. How much confidence do you have in quitting smoking successfully?

(0 = no confidence, 10 = greatest confidence) ____

D3. How difficult do you think quitting smoking is? (0 = not difficult at all, 10 = extremely difficult) _____

E. Referrals

E1. Have you ever received referrals to smoking cessation counselling/services provided by the government, universities or other institutions?

1. ☐ Yes: 0. ☐ No (jump to E4)

a. ☐ Department of Health smoking cessation counselling services 1833 183 (press 1)
b. ☐ Hospital Authority smoking cessation hotline and counselling centre 1833 183 (press 3) / 2300 7272
c. ☐ Youth Quit Line 1833 183 (press 5) / 2855 9557 / 5111 4333
d. ☐ Women Quit Line 6752 6266 / 3917 6658
e. ☐ Tung Wah Group of Hospitals 1833 183 (press 2)/ 2332 8977
f. ☐ Pok Oi Hospital 1833 183 (press 4) / 2607 1222
g. ☐ United Christian Nethersole Community Health Service 3156 9012 / 2344 3019
h. ☐ Christian Family Service Centre 2191 2887
o. ☐ Others: ____

E2. What smoking cessation counselling/services are you currently receiving?

N. ☐ Currently not receiving any smoking cessation counselling/services
a. ☐ Department of Health smoking cessation counselling services 1833 183 (press 1)
b. ☐ Hospital Authority smoking cessation hotline and counselling centre 1833 183 (press 3) / 2300 7272
c. ☐ Youth Quit Line 1833 183 (press 5) / 2855 9557 / 5111 4333
d. ☐ Women Quit Line 6752 6266 / 3917 6658
e. ☐ Tung Wah Group of Hospitals 1833 183 (press 2)/ 2332 8977
f. ☐ Pok Oi Hospital 1833 183 (press 4) / 2607 1222
g. ☐ United Christian Nethersole Community Health Service 3156 9012 / 2344 3019
h. ☐ Christian Family Service Centre 2191 2887
o. ☐ Others: ____

E3. If you have received or are currently receiving smoking cessation counselling/services, what kind of services have you used? (Can select multiple options)

a. ☐ Telephone smoking cessation counselling (frequency: ___)
b. ☐ Face-to-face counselling (frequency: ___)
c. ☐ Group discussion smoking cessation counselling (frequency: ___)
d. ☐ Smoking cessation drug therapy (frequency: ___)
e. ☐ Nicotine replacement therapy (frequency: ___)
f. ☐ Acupuncture therapy (frequency: ___)
g. ☐ Others: _____________ (frequency: ___)

E3a. How much do you think the above smoking cessation services have helped you?

4. ☐ Extremely helpful 2. ☐ Helpful
3. ☐ Very helpful 1. ☐ Quite helpful
0. ☐ Not helpful at all (Why does it not help? ____)

E3b. Would you continue to use the above smoking cessation services?

0. ☐ Yes 1. ☐ No ***(jump to E4)***

E3c. Why not?

a. ☐ Too busy b. ☐ Not interested c. ☐ No use d. ☐ Time cannot be matched e. ☐ Inconvenient venue
o. ☐ Others: ______________________________

E4. Now we can refer you to smoking cessation counselling/services to help you quit smoking and improve your health condition. Which of the following smoking cessation counselling/services would you like to be referred to?

a. ☐ Youth Quit Line 1833 183 (press 5) / 2855 9557 / 5111 4333
b. ☐ Women Quit Line 6752 6266 / 3917 6658
c. ☐ Tung Wah Group of Hospitals 1833 183 (press 2)/ 2332 8977
d. ☐ Pok Oi Hospital 1833 183 (press 4) / 2607 1222 (o. Are you on anticoagulant? ☐ Yes ☐ No)
e. ☐ Continue using current smoking cessation services
N. ☐ Not needed for now (Reason why client does not want to be referred is ____________________________)

E5. How much confidence do you have in the brief smoking cessation counselling and referrals offered by healthcare professionals can help smokers quit smoking?

(0 = no confidence; 10 = greatest confidence): _____

E6. How important do you think the brief smoking counselling and referral of healthcare professionals are to smokers to quit smoking?

(0 = not important at all; 10 = extremely important): _____

E7. How difficult do you think it is for healthcare professionals to offer brief smoking cessation counselling and referrals to smokers? (0 = not difficult at all; 10= extremely difficult): _____

E8. How effective do you think is the brief smoking cessation counselling and referrals offered by healthcare professionals in promoting smokers' attempts to quit smoking?

(0 = not effective at all; 10 = extremely effective): _____ (Why? ____________________________________)

E9. How effective do you think is the brief smoking cessation counselling and referrals offered by healthcare professionals to the success of smokers in quitting smoking?

(0= not effective at all; 10 = extremely effective): _____ (Why? ____________________________________)

F. Personal Information

F1. Gender: 1.☐ Male 2.☐ Female

F2. Age: ______

F3. Telephone number: ___________________

F4. Marital status: 1.☐ Unmarried 2.☐ Cohabited 3.☐ Married 4.☐ Separated 5.☐ Divorced 6.☐ Widowed

F5. Highest education level: (**<u>Choose one answer only</u>**)

1.☐ Not formally educated 2.☐ Primary education 3.☐ Junior secondary education 4.☐ Secondary education
5. ☐ Junior/basic diploma 6.☐ Associate degree 7.☐ Advanced diploma
8.☐ Degree or above 9. ☐ others: _________

F6. Are you studying or working now?

1.☐ Full-time student 2.☐ Full-time student part-time work 3.☐ Employed
4.☐ Self-employed 5.☐ Full-time work (employed or self-employed) part-time student
6.☐ Unemployed 7.☐ Retired 8.☐ Others：

~ End of questionnaire ~

Implementation of an evidence-based smoking cessation intervention comprising brief advice plus active referrals for smokers attending emergency departments in Hong Kong

6/12-month follow-up Questionnaire

Serial no:

Validation: agreed / refused / NA
Date: Time: Venue:
Address: [by mail]

Number of Times	1	2	3	4	5	6	7
Date							
Time							
Contact Method							
Interviewer's Name							
Follow Up Status*							

*	a	b	c	d	e	f	g
	Successful	Not available	Refused	No one answer	Directed to voice mailbox	Not connected	Number not registered

A. Smoking Status

A1. Did you smoke traditional cigarettes within the past 7 days? 1.☐ no, not even one drag 2.☐yes

A2. For how long have you quitted traditional cigarettes (not even one cigarette)?

1.☐ ________days

2.☐ quitted since ______________

(If quitted smoking <30days, jump to A4a; ≥30 days jump to A5)

A3. For how many days have you smoked traditional cigarettes within the past 7 days? ____days

A4a. How many cigarettes did you smoke per day during monday to friday within the past 30 days? ____days

A4b. How many cigarettes did you smoke per day during weekends or holidays within the past 30 days? ____days

A5. Have you tried other tobacco products within past 7 days, even one drag counts?

1. ☐ yes 2. ☐ no ***(jump to A6)***

A5a. If yes, what kind(s) of tobacco product was that? (Can select multiple options)

1. ☐ E-cigarettes 2. ☐ shisha 3. ☐ cigars 4. ☐ rolled cigarettes 5. ☐ tobacco pipes

5a. ☐heat-not-burn tobacco products (eg. IQOS) 6. ☐ Others: _________

A6. Have you tried other tobacco products (e.g. e-cigarettes, shisha, cigars, rolled cigarettes, tobacco pipes, heat-not-burn tobacco products) within past 30 days, even one drag counts?

1. ☐ yes 2. ☐ no ***(jump to A8)***

A7. How often did you use the following tobacco products?

	Never	Have tried	1 day per year or less	1 day per month or less	1 day per month	2-3 days per month	1-3 days per week	4-6 days per week	Everyday	**Minutes, Rolls, E-liquids, Cartridges…**
1. E-cigarettes: **(___day(s)/past 30 days)**	0 ☐	8 ☐	1 ☐	2 ☐	3 ☐	4 ☐	5 ☐	6 ☐	7 ☐	
2. Shisha: **(___day(s)/past 30 days)**	0 ☐	8 ☐	1 ☐	2 ☐	3 ☐	4 ☐	5 ☐	6 ☐	7 ☐	
3. Cigars: **(___day(s)/past 30 days)**	0 ☐	8 ☐	1 ☐	2 ☐	3 ☐	4 ☐	5 ☐	6 ☐	7 ☐	
4. Rolled cigarettes: **(___day(s)/past 30 days)**	0 ☐	8 ☐	1 ☐	2 ☐	3 ☐	4 ☐	5 ☐	6 ☐	7 ☐	
5. Tobacco pipes: **(___day(s)/past 30 days)**	0 ☐	8 ☐	1 ☐	2 ☐	3 ☐	4 ☐	5 ☐	6 ☐	7 ☐	
5a. Heat-not-burn tobacco products (e.g. IQOS): **(___day(s)/past 30 days)**	0 ☐	8 ☐	1 ☐	2 ☐	3 ☐	4 ☐	5 ☐	6 ☐	7 ☐	
6. Others: **(___day(s)/past 30 days)**	0 ☐	8 ☐	1 ☐	2 ☐	3 ☐	4 ☐	5 ☐	6 ☐	7 ☐	

Within past 30 days:

(only applicable to current smokers)

A8. How soon after you wake up did you smoke your first cigarette?

1.☐ Within 5 minutes (3 points) 2.☐ 6 to 30 minutes (2 points)

3.☐ 31 to 60 minutes (1 point) 4.☐ After 60 minutes (0 points)

A9. Did you find it difficult to refrain from smoking in places where it is forbidden (e.g. shopping malls, MTR, lifts)?

1.☐ Yes (1 point) 2.☐ No (0 points)

A10. Which cigarette would you find the most difficult to give up?

1.☐ The first cigarette in the morning (1 point) 2.☐ Others (0 points)

A11. Did you smoke more frequently during the first few hours after awakening than during the rest of the day?

1.☐ Yes (1 point) 2.☐ No (0 points)

A12. Would you smoke even when you are so ill that you are in bed most of the day?

1.☐ Yes (1 point) 2.☐ No (0 points)

A13. Cigarettes per day: ______

1.☐ 31 or above (3 points) 2.☐ 21 to 30 (2 points) 3.☐ 11 to 20 (1 point) 4.☐ 10 or less (0 points)

To be completed by staff: A13a. Total score: _______

A13b. Nicotine Dependency: 1.☐ Mild (0–3 points) 2.☐ Moderate (4–5 points) 3.☐ Severe (6-10 points)

B. Smoking cessation/reduction records

B1. Have you tried to **quit smoking seriously** (i.e. actively stop smoking for 24 hours or more) in the past 1 year? (Due to illness or camping/travelling is not counted)

1.☐ Yes 2.☐ No

C. Intention to quit smoking *(only applicable to current smokers)*

C1. **(If client is still smoking)** Do you plan to quit smoking now?1.☐ yes 2.☐ no (Pre-contemplation) ***(jump to D1)***

C2. When do you plan to start quitting smoking? **<u>(Choose one answer only)</u>**

0. ☐ I have stopped smoking currently (action)
1. ☐ I would quit smoking within 7 days (preparation/contemplation)
2. ☐ I would quit smoking within 1 month (preparation/contemplation)
3. ☐ I would quit smoking within 6 months (contemplation)
4. ☐ I would quit smoking after 6months (pre-contemplation)
5. ☐ Not yet decided (pre-contemplation)

C2a. 1. ☐ Decided date to quit smoking: ____________ 2. ☐ Not yet decided the exact date to quit smoking

D. Self-efficacy

D1. How important is successful quitting smoking to you? (0 = not important at all, 10 = extremely important) ____

D2. How much confidence do you have in quitting smoking successfully?
(0 = no confidence, 10 = greatest confidence) ____

D3. How difficult do you think quitting smoking is? (0 = not difficult at all, 10 = extremely difficult) _____

E. Referrals

E1. After joining this project, have our staff/Research Assistant referred you to/encourage you to continue joining any smoking cessation counselling/services provided by the government, universities or other institutions?

1. ☐ yes: 0. ☐ no

a. ☐ Department of Health smoking cessation counselling services 1833 183 (press 1)
b. ☐ Hospital Authority smoking cessation hotline and counselling centre 1833 183 (press 3) / 2300 7272
c. ☐ Youth Quit Line 1833 183 (press 5) / 2855 9557 / 5111 4333
d. ☐ Women Quit Line 6752 6266 / 3917 6658
e. ☐ Tung Wah Group of Hospitals 1833 183 (press 2)/ 2332 8977
f. ☐ Pok Oi Hospital 1833 183 (press 4) / 2607 1222
g. ☐ United Christian Nethersole Community Health Service 3156 9012 / 2344 3019
h. ☐ Christian Family Service Centre 2191 2887
o. ☐ Others: ____

E2. Which smoking cessation counselling/services are you receiving currently?

N. ☐ Currently not receiving any smoking cessation counselling/services → ***E4***
n1. ☐ Not attending or not picking up phone call from smoking cessation services (Why?______________) → ***E4***
a. ☐ Department of Health smoking cessation counselling services 1833 183 (press 1)
b. ☐ Hospital Authority smoking cessation hotline and counselling centre 1833 183 (press 3) / 2300 7272
c. ☐ Youth Quit Line 1833 183 (press 5) / 2855 9557 / 5111 4333
d. ☐ Women Quit Line 6752 6266 / 3917 6658
e. ☐ Tung Wah Group of Hospitals 1833 183 (press 2)/ 2332 8977
f. ☐ Pok Oi Hospital 1833 183 (press 4) / 2607 1222
g. ☐ United Christian Nethersole Community Health Service 3156 9012 / 2344 3019
h. ☐ Christian Family Service Centre 2191 2887
o. ☐ Others: ____

E3. If you are currently receiving smoking cessation counselling/services, what kind of services have you used? (Can select multiple options)

a. ☐ Telephone smoking cessation counselling (frequency: ___)
b. ☐ Face-to-face counselling (frequency: ___)
c. ☐ Group discussion smoking cessation counselling (frequency: ___)
d. ☐ Smoking cessation drug therapy (frequency: ___)
e. ☐ Nicotine replacement therapy (frequency: ___)
f. ☐ Acupuncture therapy (frequency: ___)
g. ☐ Others: _______________ (frequency: ___)

E3a. How much do you think the above smoking cessation services have helped you?

4. ☐ Extremely helpful 3. ☐ Very helpful 2. ☐ Helpful 1. ☐ Quite helpful
0. ☐ Not helpful at all (Why does it not help? ____)

E3b. Would you continue to use the above smoking cessation services?

0. ☐ no 1. ☐ yes → ***E5***

E3c. Why not?

a. ☐ Too busy b. ☐ Not interested c. ☐ No use d. ☐ Time cannot be matched e. ☐ Inconvenient venue
o. ☐ Others: ________________________

E4. If you are not currently receiving any smoking cessation counselling/services, do you need us to help you make an appointment or referral?

1. ☐ yes: 0. ☐ no → ***E5***
   a. ☐ Youth Quit Line 1833 183 (press 5) / 2855 9557 / 5111 4333
   b. ☐ Women Quit Line 6752 6266 / 3917 6658
   c. ☐ Tung Wah Group of Hospitals 1833 183 (press 2)/ 2332 8977
   d. ☐ Pok Oi Hospital 1833 183 (press 4) / 2607 1222 (o. Are you on anticoagulant? ☐ Yes ☐ No)

E5. How much confidence do you have in the brief smoking cessation counselling and referrals offered by healthcare professionals can help smokers quit smoking? (0 = no confidence; 10 = greatest confidence): _____

E6. How important do you think the brief smoking counselling and referral of healthcare professionals are to smokers to quit smoking? (0 = not important at all; 10 = extremely important): _____

E7. How difficult do you think it is for healthcare professionals to offer brief smoking cessation counselling and referrals to smokers? (0 = not difficult at all; 10= extremely difficult): _____

E8. How effective do you think is the brief smoking cessation counselling and referrals offered by healthcare professionals in promoting smokers' attempts to quit smoking?

(0 = not effective at all; 10 = extremely effective): _____ (Why? ____)

E9. How effective do you think is the brief smoking cessation counselling and referrals offered by healthcare professionals to the success of smokers in quitting smoking?

(0= not effective at all; 10 = extremely effective): _____ (Why? ____)

~ End of questionnaire ~
