# Supplementary material for: Evidence-based brief cessation advice plus active referral for emergency department patients who smoke: a single-arm, real-world clinical trial
Source: BMC Med. 2025 Nov 27;23:714. doi: 10.1186/s12916-025-04534-9 (PMC12751522; doi:10.1186/s12916-025-04534-9)
Supplement: Supplementary file 5 — Additional file 5. Table S1. RE-AIM Evaluation indicators. [file 12916_2025_4534_MOESM5_ESM.docx]

**Table S1. RE-AIM Evaluation indicators**

| **Outcomes/**  Indicators | **Measures** | **Level** |
| --- | --- | --- |
| **Reach:** Number, ratios, and representation of smokers meeting inclusion and exclusion criteria in the implementation research project. | | |
| Number | The number of smokers who met the inclusion and exclusion criteria, signed the consent form, and participated in the research | Individual |
| Ratio | Numerator: Number of smokers who met the inclusion and exclusion criteria, signed the consent form, and participated in the research.  Denominator: Number of smokers who met the inclusion and exclusion criteria in the research. | Individual |
| Representation | Similarity of demographics and smoking profiles between the participants recruited at baseline and participants completed the whole study at the endpoint. | Individual |
| **Efficacy:** Intervention effects at the individual level within the project. | | |
| Primary outcomes | Biochemically validated abstinence at 6-month follow-up. The self-reported quitters at the 6-month follow-up were invited to participate in a biochemical validation test, defined by an exhaled carbon monoxide level of less than 4 ppm and a salivary cotinine level of less than 10 ng/ml. | Individual |
| Secondary outcomes | (1) Biochemically validated abstinence at 12 months;  (2) Self-reported 7-day point prevalence of abstinence to all tobacco products at 6 and 12 months; and  (3) Self-reported reduction of ≥ 50% in cigarette consumption at 6 and 12 months. | Individual |
| Other outcomes | (1) Self-reported 7-day point prevalence of abstinence to traditional cigarettes at 6 and 12 months;  (2) Rate of smokers using other tobacco products at 6 and 12 months;  (3) Attempt to quit smoking at 6 and 12 months;  (4) Decreasing of nicotine dependency level at 6 and 12 months; and  (5) Promotion of the stage of readiness to quit at 6 and 12 months; | Individual |
| **Adoption:** Number, percentage, and representation of participating institutions and HCPs in the project. | | |
| Number | The number of institutions meeting the recruitment criteria and agreeing to join the project | Organization |
| Ratio | Numerator: Number of institutions meeting the recruitment criteria and agreeing to join the project;  Denominator: Number of institutions meeting the project recruitment criteria. | Organization |
| Representation | Similarity between participating institutions and all institutions meeting the recruitment criteria. | Organization |
| Number | Number of HCPs meeting the recruitment criteria and agreeing to join the project | Individual |
| Ratio | Numerator: Number of HCPs meeting the recruitment criteria and agreeing to join the project;  Denominator: Number of HCPs meeting the recruitment criteria. | Individual |
| Representation | Similarity of demographics information between the HCPs participating in the project at baseline and the HCPs completed the whole study at the endpoint. | Individual |
| **Implementation:** Degree of adherence to the intervention, local adjustments made during guideline implementation, and implementation costs. | | |
| Fidelity | Number of training session conducted in the institutions. | Organization |
| Fidelity | HCPs’ changes in the indicators below post-training and within 6 months after the completion of projects comparing to pre-training:  (1) the knowledge of the risk of smoking;  (2) attitudes towards smoking, tobacco control and smoking cessation;  (3) the self-efficacy to deliver brief smoking cessation advice and active referral; and  (4) HCPs’ satisfaction with and feedback on the training workshop. | Individual |
| Implementation costs | The operating cost of interventions, including direct operating expenses (e.g., staff members' salary and materials used for the training of counsellors), participant recruitment, and intervention delivery (e.g., boosters).  The total cost of smoking cessation services was calculated by multiplying number of successful referred participants by per person cost of the services according to previous literature.  The cost per person of providing brief advice using the AWARD model was calculated by dividing the total cost by the number of smokers. | Organization |
| **Maintenance:** Maintenance of the project after the research is completed. | | |
| Normalization | The number of institution maintenance the intervention at the endpoint of the project. | Organization |
| Fidelity | The actual number of smokers referred to smoking cessation services by trained HCPs within 6 months after the completion of projects. | Individual |
