# Supplementary material for: Evidence-based brief cessation advice plus active referral for emergency department patients who smoke: a single-arm, real-world clinical trial
Source: BMC Med. 2025 Nov 27;23:714. doi: 10.1186/s12916-025-04534-9 (PMC12751522; doi:10.1186/s12916-025-04534-9)
Supplement: Supplementary file 6 — Additional file 6. Table S2. Schedule of assessments. [file 12916_2025_4534_MOESM6_ESM.docx]

**Table S2. Schedule of assessments**

| Assessment | Time-point | | | | | |
| --- | --- | --- | --- | --- | --- | --- |
|  | Baseline | 1 week | 1 month | 3 months | 6 months | 12 months |
| Informed consent for healthcare professionals (HCPs) | **×** |  |  |  |  |  |
| Informed consent for participants | **×** |  |  |  |  |  |
| Eligibility screen for participants | **×** |  |  |  |  |  |
| Intervention initiation | **×** |  |  |  |  |  |
| Sociodemographic characteristics * | **×** |  |  |  |  |  |
| Intervention booster calls |  | **×** | **×** |  |  |  |
| Effectiveness evaluation |  |  |  |  |  |  |
| Biochemically validated abstinence |  |  |  |  | **×** | **×** |
| Self-reported 7-day point prevalence of abstinence |  |  |  |  | **×** | **×** |
| Self-reported reduction of cigarette consumption |  |  |  |  | **×** | **×** |
| Implementation evaluations |  |  |  |  |  |  |
| Number of EDs participating in this study | **×** |  |  |  |  |  |
| Participants enrolled in this study | **×** |  |  |  |  |  |
| Number of participants referred to smoking cessation services | **×** | **×** | **×** |  | **×** | **×** |
| HCPs’ knowledge of the risk of smoking | **×** |  |  | **×** | **×** |  |
| HCPs’ attitudes towards smoking, tobacco control and smoking cessation | **×** |  |  | **×** | **×** |  |
| HCPs’ self-efficacy to deliver smoking cessation advice | **×** |  |  | **×** | **×** |  |
| HCPs’ intention to deliver smoking cessation advice | **×** |  |  | **×** | **×** |  |
| HCPs’ planning to deliver smoking cessation advice | **×** |  |  | **×** | **×** |  |
| HCPs’ practices of delivering smoking cessation advice | **×** |  |  | **×** | **×** |  |
| Number of delivering brief advice and referral among healthcare professionals | **×** |  |  | **×** | **×** |  |
| Feedback Interview for HCPs |  |  |  | **×** | **×** |  |
| Feedback Interview for participants |  |  |  |  | **×** | **×** |

* Sociodemographic characteristics include age, sex, marital status, education level, and occupation.
