# Supplementary material for: Evidence-based brief cessation advice plus active referral for emergency department patients who smoke: a single-arm, real-world clinical trial
Source: BMC Med. 2025 Nov 27;23:714. doi: 10.1186/s12916-025-04534-9 (PMC12751522; doi:10.1186/s12916-025-04534-9)
Supplement: Supplementary file 7 — Additional file 7. Table S3. Comparison of baseline characteristics and smoking profiles between those who were referred to smoking cessation services in the original unmatched sample and the propensity-score matched sample. [file 12916_2025_4534_MOESM7_ESM.docx]

**Table S3.** **Comparison of baseline characteristics and smoking profiles between those who were referred to smoking cessation services in the original unmatched sample and the propensity-score matched sample.**

| Variable | Unmatched cases | |  | Matched cases | |  |
| --- | --- | --- | --- | --- | --- | --- |
|  | Referred  (n=455) | Unreferred  (n=1146) | Standardized differences | Referred  (n=396) | Unreferred  (n=792) | Standardized differences |
| Age, years, range:18-92^a^ | 47.1(14.0) | 48.8(15.4) | -0.116 | 46.4(13.6) | 47.4(14.9) | -0.070 |
| Sex |  |  |  |  |  |  |
| Male | 400(87.9) | 1043(91·0) | -0.081 | 347(87.6) | 690(87.1) | 0.012 |
| Female | 55(12.1) | 103(9.0) | 0.081 | 49(12.4) | 102(12.9) |  |
| Educational attainment ^a^ |  |  |  |  |  |  |
| Primary or below | 61(14.1) | 209(18.4) | -0.097 | 66(16.7) | 125(15.8) | 0.020 |
| Secondary | 327(75.7) | 760(66.9) | 0.162 | 286(72.2) | 552(69.7) | 0.045 |
| Tertiary or above | 44(10.2) | 167(14.7) | -0.115 | 44(11.1) | 115(14.5) | -0.085 |
| Marital status^a^ |  |  |  |  |  |  |
| Single | 125(27.8) | 328(28.8) | -0.018 | 116(29.3) | 229(28.9) | 0.007 |
| Married/cohabited | 294(65.5) | 737(64.8) | 0.012 | 253(63.9) | 514(64.9) | -0.017 |
| Separated/divorced/widowed | 30(6.7) | 72(6.3) | 0.013 | 27(6.8) | 49(6.2) | 0.020 |
| Employment status ^a^ |  |  |  |  |  |  |
| Student | 7(1.6) | 16(1.4) | 0.013 | 8(2.0) | 18(2.3) | -0.017 |
| Employed | 325(72.1) | 831(72.9) | -0.015 | 279(70.5) | 560(70.7) | -0.004 |
| Unemployed or retired | 119(26.4) | 293(25.7) | 0.013 | 109(27.5) | 214(27.0) | 0.009 |
| Daily traditional cigarette consumption, range:0-120 | 14.8(8.8) | 13.4(9.4) | 0.154 | 14.7(8.7) | 13.9(9.3) | 0.089 |
| Regular tobacco use time, years, range:0-74 | 29.5(14.5) | 30.9(15.9) | -0.092 | 28.9(14.1) | 29.6(15.2) | -0.048 |
| History of using other tobacco products ^a^ | 162(35.6) | 432(37.7) | -0.036 | 149(37.6) | 308(38.9) | -0.022 |
| Currently using other tobacco products | 44(9.7) | 135(11.8) | -0.056 | 40(10.1) | 89(11.2) | -0.029 |
| Previous ever quit attempts ^a^ | 316(69.5) | 746(65.1) | 0.077 | 276(69.7) | 553(69.8) | -0.002 |
| Previous ever quit attempts within one year ^a^ | 104(22.9) | 216(18.8) | 0.082 | 90(22.7) | 169(21.3) | 0.027 |
| Ever services used | 137(30.1) | 166(14.5) | 0.299 | 100(25.3) | 166(21.0) | 0.082 |
| Nicotine dependency by the FTND ^b^ |  |  |  |  |  |  |
| Mild, 0-3 | 186(40.9) | 586(51.2) | -0.170 | 176(44.4) | 367(46.3) | -0.031 |
| Moderate, 4-5 | 152(33.4) | 289(25.2) | 0.146 | 127(32.1) | 235(29.7) | 0.042 |
| Severe, 6-10 | 117(25.7) | 270(23.6) | 0.040 | 93(23.5) | 190(24.0) | -0.010 |
| Intention to quit ^a^ |  |  |  |  |  |  |
| Pre-contemplation | 360(79.1) | 1064(92.8) | -0.307 | 338(85.4) | 710(89.6) | -0.101 |
| Contemplation | 63(13.8) | 42(3.7) | 0.273 | 36(9.1) | 42(5.3) | 0.116 |
| Preparation | 21(4.6) | 24(2.1) | 0.107 | 13(3.3) | 24(3.0) | 0.014 |
| Action | 11(2.4) | 16(1.4) | 0.057 | 9(2.3) | 16(2.0) | 0.017 |

Note: FTND = Fagerström Test for Nicotine Dependence.

a. with missing value.
