# Supplementary material for: Evidence-based brief cessation advice plus active referral for emergency department patients who smoke: a single-arm, real-world clinical trial
Source: BMC Med. 2025 Nov 27;23:714. doi: 10.1186/s12916-025-04534-9 (PMC12751522; doi:10.1186/s12916-025-04534-9)
Supplement: Supplementary file 8 — Additional file 8. Table S4. Smokers’ demographic and smoking profile at baseline and difference among referral, self-determination and control group in the original unmatched sample. [file 12916_2025_4534_MOESM8_ESM.docx]

**Table S4. Smokers’ demographic and smoking profile at baseline and difference among referral, self-determination and control group in the original unmatched sample.**

| Variable | Unmatched | | | P value | Standardized differences | | |
| --- | --- | --- | --- | --- | --- | --- | --- |
|  | Referral  (n=1601) | Self-deter  (n=787) | Control  (n=784) |  | referral vs self-deter | referral vs control | self-deter vs control |
| Age, years, range:18-92^a^ | 48.3(15.0) | 46.7(15.9) | 47.9(16.8) | 0.083 | -0.104 | -0.073 | 0.026 |
| Sex |  |  |  | 0.133 |  |  |  |
| Male | 1443(90.1) | 688(87.4) | 700(89.3) |  | -0.069 | -0.048 | 0.022 |
| Female | 158(9.9) | 99(12.6) | 84(10.7) |  | 0.069 | 0.048 | -0.022 |
| Educational attainment ^a^ |  |  |  | <0.001 |  |  |  |
| Primary or below | 280(17.5) | 196(24.9) | 212(27.0) |  | 0.145 | -0.039 | -0.193 |
| Secondary | 1107(69.1) | 542(68.9) | 515(65.7) |  | -0.004 | 0.056 | 0.060 |
| Tertiary or above | 214(13.4) | 49(6.2) | 57(7.3) |  | -0.211 | -0.036 | 0.158 |
| Marital status ^a^ |  |  |  | <0.001 |  |  |  |
| Single | 459(28.7) | 230(29.2) | 198(25.3) |  | 0.009 | 0.071 | 0.062 |
| Married/cohabited | 1039(64.9) | 466(59.2) | 509(64.9) |  | -0.096 | -0.096 | 0.000 |
| Separated/divorced/widowed | 103(6.4) | 91(11.6) | 77(9.8) |  | 0.143 | 0.047 | -0.105 |
| Employment status ^a^ |  |  |  | 0.557 |  |  |  |
| Employed/Student | 1188(74.2) | 597(75.9) | 577(73.6) |  | 0.032 | 0.043 | 0.011 |
| Unemployed or retired | 413(25.8) | 190(24.1) | 207(26.4) |  | -0.032 | -0.043 | -0.011 |
| Daily traditional cigarette consumption, range:0-120 | 13.9(9.2) | 13(8.0) | 12.5(7.6) | 0.001 | -0.104 | 0.064 | 0.162 |
| Regular tobacco use time, years, range:0-74 | 30.5(15.6) | 29.4(16.3) | 30.3(16.8) | 0.327 | 0.016 | -0.011 | -0.027 |
| Previous ever quit attempts ^a^ | 320(20.0) | 551(70.0) | 518(66.1) | <0.001 | 0.928 | 0.069 | -0.884 |
| Nicotine dependency by the FTND ^b^ |  |  |  | <0.001 |  |  |  |
| Mild, 0-3 | 773(48.3) | 392(49.8) | 397(50.6) |  | 0.024 | -0.013 | -0.038 |
| Moderate, 4-5 | 441(27.5) | 338(42.9) | 347(44.3) |  | 0.262 | -0.023 | -0.296 |
| Severe, 6-10 | 387(24.2) | 57(7.2) | 40(5.1) |  | -0.427 | 0.070 | 0.419 |
| Intention to quit ^a^ |  |  |  | <0.001 |  |  |  |
| Pre-contemplation | 1424(88.9) | 245(31.1) | 423(54.0) |  | -1.126 | -0.394 | 0.739 |
| Contemplation | 105(6.6) | 265(33.7) | 127(16.2) |  | 0.537 | 0.324 | -0.267 |
| Preparation | 45(2.8) | 264(33.5) | 218(27.8) |  | 0.631 | 0.100 | -0.700 |
| Action | 27(1.7) | 13(1.7) | 16(2.0) |  | 0.000 | -0.018 | -0.018 |

Note: FTND = Fagerström Test for Nicotine Dependence. Referral= participants in the current study receiving the brief advice + active referral; Self-Quit = participants in the previous RCT receiving brief advice and self-determine to quit immediately or progressive; control = participants in the previous RCT receiving smoking cessation leaflet and placebo treatment.
