# Supplementary material for: Evidence-based brief cessation advice plus active referral for emergency department patients who smoke: a single-arm, real-world clinical trial
Source: BMC Med. 2025 Nov 27;23:714. doi: 10.1186/s12916-025-04534-9 (PMC12751522; doi:10.1186/s12916-025-04534-9)
Supplement: Supplementary file 10 — Additional file 10. Table S6. Sensitivity analysis using GEE, multiple imputed outcomes, and completed cases. [file 12916_2025_4534_MOESM10_ESM.docx]

The GEE was first used to obtain the primary outcome at the 6-month follow-up and secondary outcomes at each follow-up, adjusted for age, hospital, employment status, annual income level, hospitals, level of nicotine dependence, level of readiness to quit, and smoking self-efficacy at baseline. As the primary outcome was only measured at the baseline and the 6-month follow-up, repeated measures could not be performed since all participants were smokers at the baseline. Therefore, the covariance estimator in the robust estimation of the first-order autoregressive model was further performed for the self-reported 7-day PPA by excluding baseline values and for the level of readiness to quit at all time points.

**Table S6. Sensitivity analysis using GEE, multiple imputed outcomes, and completed cases.**

| Variable | GEE Adjusted OR^a,d^ (95% CI) ^c^ | P value | MI Adjusted OR^b,d^ (95% CI) | P value | CC Adjusted OR^c,d^  (95% CI) | P value | |
| --- | --- | --- | --- | --- | --- | --- | --- |
| Biochemically validated abstinence | | | | | | |  |
| 6m | 1.58(1.15, 2.62) | 0.034 | 1.83(1.25, 2.66) | 0.002 | 2.03(1.12, 3.70) | 0.020 | |
| 12m | 2.65(1.65, 4.27) | <0.001 | 2.27(1.39, 3.73) | 0.001 | 3.09(1.78, 5.37) | <0.001 | |
| Self-reported 7-day PPA of all tobacco products | | | | | | |  |
| 6m | 1.75(1.25, 2.45) | 0.001 | 1.58(1.11, 2.26) | 0.012 | 1.68(1.13, 2.50) | 0.010 | |
| 12m | 1.40(1.04, 1.88) | 0.028 | 1.28(1.12, 1.45) | 0.008 | 1.61(1.15, 2.24) | 0.005 | |
| Self-reported 7-day PPA of traditional cigarette | | | | | | |  |
| 6m | 1.18(0.87, 1.59) | 0.288 | 1.14(0.83, 1.57) | 0.409 | 1.26(0.88, 1.79) | 0.201 | |
| 12m | 1.25(0.94, 1.67) | 0.119 | 1.21(0.89, 1.63) | 0.225 | 1.26(0.90, 1.76) | 0.176 | |
| Self-reported reduction of ≥ 50% in cigarette consumption ^e^ | | | | | | |  |
| 6m | 1.91(1.40, 2.61) | <0.001 | 1.69(1.22, 2.25) | 0.002 | 1.77(1.23, 2.56) | 0.002 | |
| 12m | 1.52(1.14, 2.04) | 0.005 | 1.90(1.33, 2.72) | <0.001 | 1.78(1.21, 2.63) | 0.004 | |
| Attempt to quit smoking ^e^ | | | | | | |  |
| 6m | 1,54(1.27, 1.88) | <0.001 | 1.86(1.44, 2.41) | <0.001 | 1.71(1.29, 2.26) | <0.001 | |
| 12m | 1.49(1.23, 1.81) | <0.001 | 1.84(1.45, 2.23) | <0.001 | 1.78(1.38, 2.31) | <0.001 | |
| Currently using other tobacco products | | | | | | |  |
| 6m | 0.79(0.54, 1.13) | 0.192 | 0.72(0.42, 1.23) | 0.224 | 0.68(0.35, 1.32) | 0.252 | |
| 12m | 0.77(0.57, 1.03) | 0.080 | 0.70(0.39, 1.23) | 0.213 | 0.59(0.32, 1.10) | 0.095 | |
| FTND decreasing ^e,f^ | | | | | | |  |
| 6m | 1.32(0.96, 1.84) | 0.092 | 1.08(0.84, 1.40) | 0.542 | 1.07(0.81, 1.42) | 0.649 | |
| 12m | 1.25(0.94, 1.66) | 0.121 | 1.09(0.84, 1.41) | 0.523 | 1.05(0.80, 1.39) | 0.722 | |
| TTM stage promotion ^e,f^ | | | | | | |  |
| 6m | 3.50(2.17, 5.66) | <0.001 | 2.65(1.93, 3.65) | <0.001 | 2.54(1.81, 3.56) | <0.001 | |
| 12m | 2.85(2.16, 3.77) | <0.001 | 1.89(1.47, 2.43) | <0.001 | 1.78(1.35, 2.35) | <0.001 | |

Note:

a. Generalized Estimating Equation Model was conducted considering participants lost to follow-up were assumed to be active smokers with no changes in their habits at baseline.

b. Multiple logistic regression was conducted using multiple imputed outcomes with outcomes of participants lost to follow-up or refused to participate in the validation tests.

c. Multiple logistic regression was conducted using completed cases.

d, Estimates adjusting for age, gender, education level, marital status, employment, cigarette consumption per day, regular tobacco use time, nicotine dependence level by FTND, stage of readiness to quit, quit attempt, referral history, other tobacco use, psychological perspectives on importance, confidence and difficulty of quitting smoking at baseline.

e. Quitters were excluded.

f. Compared to that at baseline.
