# Supplementary material for: Evidence-based brief cessation advice plus active referral for emergency department patients who smoke: a single-arm, real-world clinical trial
Source: BMC Med. 2025 Nov 27;23:714. doi: 10.1186/s12916-025-04534-9 (PMC12751522; doi:10.1186/s12916-025-04534-9)
Supplement: Supplementary file 11 — Additional file 11. Table S7. Similarity of demographics and smoking profiles between the participants recruited at baseline and participants completed the whole study at the endpoint. [file 12916_2025_4534_MOESM11_ESM.docx]

**Table S7. Similarity of demographics and smoking profiles between the participants recruited at baseline and participants completed the whole study at the endpoint.**

| Variable | Baseline  (N=1601) | Complete the study  (N=1154) | P values |
| --- | --- | --- | --- |
| Successful referred to smoking cessation services | 455(28.4) | 352(30.5) | 0.236 |
| Age, years, range:18-92^a^ | 48.3(15.0) | 47.9(14.8) | 0.473 |
| Sex |  |  | 0.404 |
| Male | 1443(90.1) | 1051(91.1) |  |
| Female | 158(9.9) | 103(8.9) |  |
| Educational attainment ^a^ |  |  | 0.729 |
| Primary or below | 270(17.2) | 187(16.6) |  |
| Secondary | 1087(69.3) | 779(69.0) |  |
| Tertiary or above | 211(13.5) | 163(14.4) |  |
| Marital status^a^ |  |  | 0.671 |
| Single | 453(28.6) | 310(27.1) |  |
| Married/cohabited | 1031(65.0) | 763(66.6) |  |
| Separated/divorced/widowed | 102(6.4) | 73(6.4) |  |
| Employment status ^a^ |  |  | 0.219 |
| Student | 23(1.4) | 19(1.7) |  |
| Employed | 1156(72.7) | 863(75.3) |  |
| Unemployed or retired | 412(25.9) | 264(23.0) |  |
| Daily traditional cigarette consumption, range:0-120 | 13.8(9.3) | 13.9(9.3) | 0.841 |
| Regular tobacco use time, years, range:0-74 | 30.5(15.5) | 30.1(15.4) | 0.568 |
| History of using other tobacco products ^a^ | 594(37.1) | 431(37.3) | 0.895 |
| Currently using other tobacco products | 179(11.2) | 134(11.6) | 0.725 |
| Previous ever quit attempts ^a^ | 1062(66.3) | 774(67.1) | 0.685 |
| Previous ever quit attempts within one year ^a^ | 320(20.0) | 925(80.2) | 0.926 |
| Ever services used | 303(18.9) | 225(19.5) | 0.707 |
| Nicotine dependency by the FTND ^b^ |  |  | 0.924 |
| Mild, 0-3 | 772(48.3) | 548(47.5) |  |
| Moderate, 4-5 | 441(27.5) | 320(27.8) |  |
| Severe, 6-10 | 387(24.2) | 285(24.7) |  |
| Intention to quit ^a^ |  |  | 0.818 |
| Pre-contemplation | 1422(88.9) | 1015(88.0) |  |
| Contemplation | 105(6.6) | 84(7.3) |  |
| Preparation | 45(2.8) | 32(2.8) |  |
| Action | 17(1.7) | 23(2.0) |  |
