# Supplementary material for: Evidence-based brief cessation advice plus active referral for emergency department patients who smoke: a single-arm, real-world clinical trial
Source: BMC Med. 2025 Nov 27;23:714. doi: 10.1186/s12916-025-04534-9 (PMC12751522; doi:10.1186/s12916-025-04534-9)
Supplement: Supplementary file 12 — Additional file 12. Table S8. Similarity between participating institutions and all institutions meeting the recruitment criteria. [file 12916_2025_4534_MOESM12_ESM.docx]

**Table S8. Similarity between participating institutions and all institutions meeting the recruitment criteria.**

| Variable | N(%) or Mean (SD) | | P value |
| --- | --- | --- | --- |
|  | Enrolled institution | All institution |  |
| Number of district of Hospitals |  |  | 0.522 |
| Hong Kong Island | 2(50) | 4(22.2) |  |
| Kowloon | 1(25.0) | 8(43.3) |  |
| New Territories | 1(25.0) | 6(33.3) |  |
| Number of full-time doctors in EDs | 40.0(6.5) | 31.7(8.5) | 0.087 |
| Number of full-time nurses in EDs | 91.3(24.9) | 77.4(23.8) | 0.310 |
| Number of annual EDs patients visiting^a^ | 111.6(16.4) | 91.1(31.5) | 0.228 |
| Waiting time for patients triaged as level 4 ^b^ | 2.4(1.0) | 1.9(0.8) | 0.259 |
| Waiting time for patients triaged as level 5 ^b^ | 3.0(1.1) | 2.2(0.9) | 0.159 |

**Notes:** a: thousand person time, b: hours. Data were extracted from the Services of the accident and emergency departments of public hospitals. <https://www.info.gov.hk/gia/general/202104/21/P2021042000635.htm> , and <https://www.info.gov.hk/gia/general/202310/18/P2023101800323.htm>.
