# Supplementary material for: Evidence-based brief cessation advice plus active referral for emergency department patients who smoke: a single-arm, real-world clinical trial
Source: BMC Med. 2025 Nov 27;23:714. doi: 10.1186/s12916-025-04534-9 (PMC12751522; doi:10.1186/s12916-025-04534-9)
Supplement: Supplementary file 13 — Additional file 13. Table S9. Similarity of demographics information between the HCPs participating in the project at baseline and the HCPs completed the whole study at the endpoint. [file 12916_2025_4534_MOESM13_ESM.docx]

**Table S9. Similarity of demographics information between the HCPs participating in the project at baseline and the HCPs completed the whole study at the endpoint.**

| Variable | N(%) or Mean (SD) | | P value |
| --- | --- | --- | --- |
|  | Baseline  (N=76) | Completed the study  (N=35) |  |
| Age, ranged 21-56 | 31.0(8.1) | 32.4(7.7) | 0.433 |
| Sex |  |  | 0.804 |
| Male | 20(26.3) | 10(28.6) |  |
| Female | 56(74.7) | 25(71.4) |  |
| Smoking status |  |  | 1 |
| Never smoked | 76(100.0) | 35(100.0) |  |
| Affiliation |  |  | 0.177 |
| PYNEH | 13(17.1) | 4(11.4) |  |
| TKOH | 12(15.8) | 12(34.3) |  |
| TMH | 14(18.2) | 5(14.3) |  |
| UCH | 37(48.7) | 14(40/0) |  |
| Position |  |  | 0.198 |
| RN/EN | 65(85.5) | 25(71.4) |  |
| APN/WM | 9(11.9) | 8(22.9) |  |
| NC/NO | 2(2.6) | 2(5.7) |  |

Note: PYNEH, Pamela Youde Nethersole Eastern Hospital.TKOH, Tseung Kwan O Hospital. TMH, Tuen Mun Hospital. UCH, United Christian Hospital. RN, registered nurse. EN, emergency nurse. APN, Advanced Practice Nurse. WM, Ward Manager. NC, Nurse Consultant. NO, Nurse Officer. Data were extracted from the Services of the accident and emergency departments of public hospitals. <https://www.info.gov.hk/gia/general/202104/21/P2021042000635.htm> , and <https://www.info.gov.hk/gia/general/202310/18/P2023101800323.htm>.
