# Supplementary material for: Evidence-based brief cessation advice plus active referral for emergency department patients who smoke: a single-arm, real-world clinical trial
Source: BMC Med. 2025 Nov 27;23:714. doi: 10.1186/s12916-025-04534-9 (PMC12751522; doi:10.1186/s12916-025-04534-9)
Supplement: Supplementary file 14 — Additional file 14. Table S10. Healthcare professionals’ knowledge about the risks of smoking, attitudes towards smoking, self-efficacy to deliver brief smoking cessation advice and active referral, and satisfaction with and feedback on the training workshop at pre-training, post-training, 6-month follow-ups after the research completed. [file 12916_2025_4534_MOESM14_ESM.docx]

**Table S10. Healthcare professionals’ knowledge about the risks of smoking, attitudes towards smoking, self-efficacy to deliver brief smoking cessation advice and active referral, and satisfaction with and feedback on the training workshop at pre-training, post-training, 6-month follow-ups after the research completed.**

|  | Pre-training  (N=76) |  | Post-training  (N=76) | |  | 6-month  (N=35) | |
| --- | --- | --- | --- | --- | --- | --- | --- |
| Variables | N (%) |  | N (%) | P value ^b^ |  | N (%) | P value ^b^ |
| **Knowledge about the risks of smoking** | **9.4(1.7)** |  | **10.8(1.5)** | **<0.001** |  | **10.1(1.9)** | **0.099** |
| 1. No matter how long a person smokes, quitting smoking is never too late | 74(97.4) |  | 75(98.7) | 0.564 |  | 32(91.4) | 0.577 |
| 2. Among every two smokers, at least one would die prematurely because of smoking | 50(65.8) |  | 75(98.7) | <0.001 |  | 33(94.3) | 0.013 |
| 3. Two in three who started heavy smoking in young age dies prematurely because of smoking | 50(66.7) |  | 71(93.4) | <0.001 |  | 31(88.6) | 0.046 |
| 4. NRT patch & gum can increase the success rate of quitting smoking | 56(73.7) |  | 70(63.1) | 0.018 |  | 31(88.6) | 0.070 |
| 5. Second hand smoke intensifies outdoor air pollution and is harmful to health | 72(96.0) |  | 75(98.7) | 0.157 |  | 32(91.4) | 0.705 |
| 6. After someone smoking in a house, the residual chemicals left in the environment, for example, clothes, wall, furnitures, would damage the health of infants and children | 76(100.0) |  | 76(100.0) | 1.000 |  | 34(97.1) | 0.317 |
| 7. Use of e-cigarette is harmful to human health | 71(93.4) |  | 75(98.7) | 0.046 |  | 30(85.7) | 0.201 |
| 8. Use of e-cigarette is allowed in non-smoking area | 7(9.2) |  | 51(67.1) | 0.018 |  | 27(77.1) | 0.875 |
| 9. Shisha is less harmful than traditional rolled cigarettes | 48(63.2) |  | 57(75.0) | 0.481 |  | 21(60.0) | 0.756 |
| 10. Shisha contains less nicotine than traditional rolled cigarettes | 31(40.8) |  | 53(70.7) | 0.011 |  | 15(42.9) | 0.362 |
| 11. Heat-not-burn tobacco products can be addictive | 48(63.2) |  | 71(93.4) | <0.001 |  | 34(97.1) | 0.003 |
| 12. Heat-not-burn tobacco products is harmful to human health | 63(82.9) |  | 74(97.4) | 0.005 |  | 34(97.1) | 0.102 |
| **Attitudes towards smoking** | **9.1(2.2)** |  | **9.8(2.0)** | **<0.001** |  | **9.1(2.5)** | **0.238** |
| 1. I would proactively advise friends to quit smoking | 67(88.2) |  | 71(93.4) | 0.102 |  | 28(80.0) | 0.705 |
| 2. I would ask people not to smoke around me | 65(85.5) |  | 68(89.5) | 0.083 |  | 26(74.3) | 0.180 |
| 3. I would remind others that they are not allowed to smoke in non-smoking area | 53(69.7) |  | 63(82.9) | 0.008 |  | 25(71.4) | 0.366 |
| 4. I agree to expand non-smoking areas | 73(97.3) |  | 73(97.3) | 1.000 |  | 31(88.6) | 0.414 |
| 5. I agree to ban exhibiting cigarette products in shops, newspaper stands and other places | 64(84.2) |  | 70(92.1) | 0.014 |  | 29(82.9) | 0.564 |
| 6. I concur the government to increase funding for smoking cessation services | 69(90.8) |  | 69(90.8) | 1.000 |  | 29(82.9) | 0.083 |
| 7. I concur raising tobacco tax | 57(75.0) |  | 61(81.3) | 0.102 |  | 31(88.6) | 0.414 |
| 8. I concur implementing full warning packaging (all tobacco products must follow standardized packaging, no brand logos can be shown, brand name can only be displayed in designated colour, fonts and position) | 62(81.6) |  | 71(93.4) | 0.007 |  | 29(82.9) | 0.527 |
| 9. I agree to ban selling e-cigarette comprehensively | 59(77.6) |  | 66(86.8) | 0.008 |  | 29(82.9) | 0.102 |
| 10. I agree to ban selling heat-not-burn tobacco products comprehensively | 60(78.9) |  | 68(89.5) | 0.005 |  | 29(82.9) | 0.059 |
| 11. I agree to ban selling tobacco comprehensively | 62(81.6) |  | 68(89.5) | 0.014 |  | 32(91.4) | 0.317 |
| 13. What is the effectiveness of providing brief counselling and smoking cessation services referral to smokers in smoking cessation (mean±SD) | 4.3(2.0) |  | 5.3(2.1) | <0.001^c^ |  | 5.0(2.4) | 0.342 ^c^ |
| 14. What is the effectiveness of providing brief counselling and smoking cessation services referral to smokers in helping them to quit smoking (mean±SD) | 4.7(2.2) |  | 5.5(2.2) | <0.001^c^ |  | 5.1(2.3) | 0.578 ^c^ |
| **Self-efficacy to deliver brief smoking cessation advice and active referral** | **16.6(2.8)** |  | **17.3(4.3)** | **<0.034^c^** |  | **17.8(2.3)** | **0.458** |
| 1. How much confidence in providing brief smoking cessation counselling and referring smokers to smoking cessation services | 4.3(2.3) |  | 5.4(2.0) | <0.001^c^ |  | 4.6(2.2) | 0.860 ^c^ |
| 2. How important do you think brief smoking cessation counselling and referrals are in helping smokers quit smoking | 5.6(2.1) |  | 6.1(1.9) | 0.002 ^c^ |  | 5.7(2.3) | 0.286 ^c^ |
| 3. How difficult do you think in providing brief smoking cessation counselling and referral | 6.7(2.0) |  | 5.8(2.0) | 0.002 ^c^ |  | 6.3(2.5) | 0.283 ^c^ |
| **Satisfaction with and feedback on the training workshop** |  |  |  |  |  |  |  |
| 1. The content of the training workshop is enriching | - |  | 63(85.1)^d^ |  |  | - |  |
| 2. The environment and facilities of the training workshop is comprehensive | - |  | 61(82.4) ^d^ |  |  | - |  |
| 3. The time arrangement of the training workshop is ideal | - |  | 68(91.9) ^d^ |  |  | - |  |
| 4. The training workshop helped me to develop independent and critical thinking ability | - |  | 48(64.9) ^d^ |  |  | 19(59.4) | 0.208 |
| 5. The training workshop helped me to improve communication skills | - |  | 45(60.8) ^d^ |  |  | 18(56.3) | 0.259 |
| 6. The training workshop helped me to improve problem-solving abilities | - |  | 42(56.8) ^d^ |  |  | 18(56.3) | 0.901 |
| 7. The training workshop helped me to grasp smoking cessation councelling techniques | - |  | 47(63.5) ^d^ |  |  | 26(81.3) | 0.032 |
| 8. The training workshop made me understand the advantages of a smoke-free lifestyle | - |  | 55(75.3) ^d^ |  |  | 25(78.1) | 0.795 |
| 9. The training workshop has eliminated the obstacles I encountered when offering brief smoking cessation advise and referral | - |  | 43(48.1) ^d^ |  |  | 15(46.9) | 0451 |
| 10. The training workshop helped me understand the harm of smoking | - |  | 68(91.9) ^d^ |  |  | 27(84.4) | 0279 |
| 11. The training workshop helped me grasp the information related to the smoking cessation counselling/services offered by other organizations | - |  | 51(68.9) ^d^ |  |  | 24(75.0) | 0.391 |
| 12. The training workshop raised my confidence in offering brief smoking cessation advice and referral | - |  | 53(71.6) ^d^ |  |  | 12(37.5) | 0.002 |
| 13. The training workshop helped me to offer brief smoking cessation advice and referral in my working environment | - |  | 58(78.4) ^d^ |  |  | 18(56.3) | 0.110 |
| 14. The training workshop helped me to offer brief smoking cessation advice and referral in the community | - |  | 53(71.6) ^d^ |  |  | 16(60.0) | 0.104 |
| 15. The information taught by the training workshop tutor is very suitable for me | - |  | 57(77.0) ^d^ |  |  | - |  |
| 16. The training workshop tutor knows very well about smoking cessation counselling | - |  | 62(83.8) ^d^ |  |  | - |  |
| 17. The content and activities of the training workshop have fully met my expectations and targets | - |  | 51(69.9) ^d^ |  |  | - |  |
| 18. I would introduce this training workshop to friends/colleagues | - |  | 48(64.9) ^d^ |  |  | - |  |
| 19. Overall, I am satisfied with this training workshop | - |  | 61(83.6) ^d^ |  |  | - |  |
| 20. If there would be a new training workshop, would you join | - |  | 37(52.1) ^d^ |  |  | - |  |

Note: a. Proportion of HCPs that agreed the description of items.

b. Comparing to that pre-training using Related-Samples Wilcoxon Signed Rank Test.

c. Comparing to that pre-training using Paired Samples Statistics.

d. The denominator was 74.

e. Comparing to that post-training using Related-Samples Wilcoxon Signed Rank Test.
