# Supplementary material for: Evidence-based brief cessation advice plus active referral for emergency department patients who smoke: a single-arm, real-world clinical trial
Source: BMC Med. 2025 Nov 27;23:714. doi: 10.1186/s12916-025-04534-9 (PMC12751522; doi:10.1186/s12916-025-04534-9)
Supplement: Supplementary file 15 — Additional file 15. Cost effectiveness analysis. [file 12916_2025_4534_MOESM15_ESM.docx]

**Cost effectiveness analysis**

We evaluated the of brief advice plus active referral smoking cessation intervention targeted at emergency department patients. The analysis examines the incremental costs and outcomes, including quit rates and quality-adjusted life years (QALYs), to determine whether the active referral represents a cost-effective use of healthcare resources. the calculation details were demonstrated as below:

A total of 1601 participants were categorized into the referred group (455 participants, quit rate 6.1%) and unreferred group (1146 participants, quit rate 3.2%), with an incremental biochemical validated 7-days PPA rate of 2.9%. The incremental Effectiveness (ΔE) was calculated as follows:

$$\Delta E=E_{referred}-E_{unreferred}=0.061-0.032=0.029$$

**Cost**

The total cost for personnel training for healthcare professionals was 1,079.12 USD (HK$ 8,400), and the cost of intervention delivery and equipment was 13,225.06 USD (HK$ 102,945.25) for both referred and unreferred groups. Participants in the referred group were actively referred to 10 free smoking cessation services funded by the Government or NGOs. The total cost of smoking cessation services was calculated based on the number of successfully referred participants and the per-person cost of the services, as reported in the literature (107.01 USD per person). This cost was 48,690.92 USD, which occurred only in the referred group. Thus, the total cost for the referred group was 55,843.01 USD, while the unreferred group incurred 7,152.09 USD. The incremental cost per participant (ΔC) was 116.47 USD/person, calculated as follows:

$$\Delta C=\frac{C_{referred}}{No. referred participants}-\frac{C_{unreferred}}{No. unreferred participants}=\frac{55,843.01}{455}-\frac{7,152.09}{1146}=116.49 USD/person$$

**Quality-Adjusted Life Years (QALYs)**

Although we did not collect the index that could be used to estimate the Quality-Adjusted Life Year (QALY) in this study. a recent evaluation study on smoking cessation interventions in emergency departments reported a lifetime QALY gain of 0.101 per quitter. Using this value, the incremental lifetime QALY (ΔQALY) was 0.002929, calculated as follows:

$$\Delta QALY=\Delta E\times lifetime QALY per quitter =0.029 \times0.101=0.002929$$

**Incremental Cost-Effectiveness Ratios (ICERs)**

Given the indicators above, the incremental cost per successful quitter was 4,016.90 USD, while the incremental cost-effectiveness ratio (ICER) was 39,771.25 USD per QALY, calculated as follows:

$${ICER}_{quit}=\frac{\Delta C}{\Delta E}=\frac{116.49}{0.029}=4,016.90 USD/\mathrm{quitter}$$

$${ICER}_{QALY}=\frac{\Delta C}{\Delta QALY}=\frac{116.49}{0.002929}=39,771.25 \mathrm{USD}/\mathrm{QALY}$$

**Sensitivity Analysis**

To test the robustness of the results, we conducted the sensitivity analysis assuming the changes in QALY gains of ± 10%. The ICER with the impact of 10% increase in QALY was 36,155.68 USD per QALY, while the ICER with the impact of 10% decrease in QALY was 44,190.28 USD per QALY. Sensitivity analyses demonstrated the intervention's robustness to variations in effectiveness.

$${ICER}_{QALY+10\%}=\frac{\Delta C}{\Delta QALY}=\frac{116.49}{0.002929\times1.1}=36,155.68 USD/\mathrm{QALY}$$

$${ICER}_{QALY-10\%}=\frac{\Delta C}{\Delta QALY}=\frac{116.49}{0.002929\times0.9}=44,190.28 USD/\mathrm{QALY}$$

The incremental cost per QALY for the intervention is 39,771.25 USD. The GDP per capita in Hong Kong was about 54107 USD in 2024. As suggested by the World Health Organization, the active referral was considered cost-effective compared to the unreferred as the ICER was lower than 1 to 3 times Hong Kong’s gross domestic product per capita. Future research should assess the scalability of this intervention and explore strategies to further optimize costs and improve effectiveness.
